# Development of Environmentally Responsive Self-Emulsifying System Containing Copaiba Oil-Resin for Leishmaniasis Oral Treatment

**DOI:** 10.3390/pharmaceutics15082127

**Published:** 2023-08-12

**Authors:** Mariana Carla de Oliveira, Rodolfo Bento Balbinot, Mônica Villa Nova, Renato Sonchini Gonçalves, Danielle Lazarin Bidóia, Wilker Caetano, Celso Vataru Nakamura, Marcos Luciano Bruschi

**Affiliations:** 1 Postgraduate Program in Pharmaceutical Sciences, Laboratory of Research and Development of Drug Delivery Systems, Department of Pharmacy, State University of Maringa, Av. Colombo 5790, Maringa 87020-900, PR, Brazil; mcarladeoliveira@gmail.com (M.C.d.O.); monica.villano@gmail.com (M.V.N.); 2 Postgraduate Program in Biological Sciences, Laboratory of Technological Innovation in the Development of Pharmaceuticals and Cosmetics, Department of Health Basic Sciences, State University of Maringa, Av. Colombo 5790, Maringa 87020-900, PR, Brazil; rodolfobalbinot@hotmail.com (R.B.B.); dlbidoia@gmail.com (D.L.B.); cvnakamura@uem.br (C.V.N.); 3 Research Nucleus in Photodynamic Systems and Nanomedicine, Department of Chemistry, State University of Maringa, Av. Colombo 5790, Maringa 87020-900, PR, Brazil; rsonchini@gmail.com (R.S.G.); wcaetano@uem.br (W.C.)

**Keywords:** *Copaiba reticulata*, SEDDS, stimulus-responsive, emulsion systems, natural products, drug delivery

## Abstract

Leishmaniasis is a disease caused by protozoa species of the *Leishmania* genus, and the current treatments face several difficulties and obstacles. Most anti-leishmanial drugs are administered intravenously, showing many side effects and drug resistance. The discovery of new anti-leishmanial compounds and the development of new pharmaceutical systems for more efficient and safer treatments are necessary. Copaiba oil-resin (CO) has been shown to be a promising natural compound against leishmaniasis. However, CO displays poor aqueous solubility and bioavailability. Self-emulsifying drug delivery systems (SEDDS) can provide platforms for release of hydrophobic compounds in the gastrointestinal tract, improving their aqueous solubilization, absorption and bioavailability. Therefore, the present work aimed to develop SEDDS containing CO and Soluplus^®^ surfactant for the oral treatment of leishmaniasis. The design of the systems was accomplished using ternary phase diagrams. Emulsification and dispersion time tests were used to investigate the emulsification process in gastric and intestinal environments. The formulations were nanostructured and improved the CO solubilization. Their in vitro antiproliferative activity against promastigote forms of *L. amazonensis* and *L. infantum*, and low in vitro cytotoxicity against macrophages were also observed. More studies are necessary to determine effectiveness of SOL in these systems, which can be candidates for further pharmacokinetics and in vivo investigations.

## 1. Introduction

Leishmaniasis is a parasitic disease caused by protozoa of the genus *Leishmania* and it is considered a neglected tropical disease displaying different clinical conditions ranging from cutaneous and/or mucocutaneous forms to visceral leishmaniasis [1,2]. This complex disease is dependent on the immunological status of the patient and the species of Leishmania involved. The visceral leishmaniasis is the most severe manifestation and almost always fatal if not properly treated [1].

The World Health Organization (WHO) recommends the pentavalent antimonial compounds n-methyl glucamine antimoniate (Glucantime^®^) and sodium stibogluconate (Pentostan^®^) as the first-choice treatment. However, they are not effective in some cases, causing side effects such as cardiac and renal toxicity. Amphotericin B is the second-choice drug for the treatment, but displays high toxicity. Liposomal formulations containing amphotericin B (Ambisome^®^) have been shown to be less toxic, but their high cost is a barrier to their use, mainly in developing countries. Furthermore, *Leishmania* resistant to amphotericin B and pentavalent antimonials have already been found. Methylphosine has been used as a third line of treatment, and the advantage of this medicine is that it is an oral treatment, which is the most accessible and comfortable route of administration for the patient; however, it displays teratogenicity and can cause gastrointestinal discomfort [3,4,5,6,7].

Therefore, the current treatment of leishmaniasis faces some obstacles and difficulties, requiring the search for new, more effective and safe therapeutic agents, as well as the development of new formulations. Plants are one of the best sources for researching new therapeutic compounds, many of which are used in folk medicine to treat various illnesses [8,9]. Copaiba oil-resin (CO) has been used in traditional medicine since the 16th century, mainly as an anti-inflammatory, antibacterial agent and for the treatment and healing of wounds [10,11,12].

Recent studies have demonstrated important anti-Leishmania activities of CO, making it a promising therapeutic agent for the treatment of leishmaniasis [13,14]. CO from *Copaifera reticulata* Ducke demonstrated high biological activity against the promastigote and amastigote forms of *L. amazonensis* [15]. Diterpene acids of CO caused structural alterations in the protozoa of Leishmania, such as disruption of the plasma membrane with loss of intracellular contents, and alterations in mitochondria [16]. Furthermore, the anti-*Leishmania* action of CO has been mainly attributed to the synergistic effect of the natural mixture of diterpene acids and sesquiterpenes [17].

Per oral is one of the most common and safest administration routes of medicines. A drug’s absorption from the gastrointestinal tract is an important factor, with the speed and extent of adsorption mainly determined by the dissolution rate, solubility and permeability of the drug [18,19,20,21]. The enterocytes constitute a lipid barrier to the drug absorption in the gastrointestinal tract, and hydrophobic bioactive compounds can cross it more easily [22]. Thus, due to its chemical composition, CO contains some substances that can permeate, but are poorly soluble in the gastrointestinal tract environment, leading to low bioavailability [22,23,24,25].

Drug delivery systems based on lipid compounds (lipid-based delivery systems) have gained prominence in recent years, due to their ability to improve the solubility and, consequently, the bioavailability of poorly soluble bioactive agents in gastrointestinal fluids. These systems include vesicular systems (e.g., liposomes), particulate lipid systems (e.g., solid lipid nanoparticles and nanostructured lipid systems) and emulsifying systems (e.g., emulsion and self-emulsifying systems—SEDDS) [26,27]. SEDDS have been shown to improve the bioavailability of bioactive compounds of natural origin, such as cannabidiol, curcumin and quercetin [28].

For SEDDS, the use of surfactants is important for decreasing the interfacial tension between the oil and aqueous phases, allowing an intimate contact between the phases with greater dispersion stability [19,27]. Currently, the polyvinyl caprolactam-polyvinyl acetate-polyethylene glycol graft co-polymer (Soluplus^®^; SOL) is a surfactant of great pharmaceutical interest [29,30]. Due to its amphiphilic chemical structure, SOL can solubilize components with low aqueous solubility [31]. When dispersed in a medium with hydrophilic and hydrophobic phases, the hydrophobic segments of SOL (polyvinyl caprolactam and polyvinyl acetate) can stay in contact with the oil phase, while the hydrophilic segment, constituted by polyethylene, is more localized in the hydrophilic phase. The emulsions formed with SEDDS are most often of the oil-in-water type where the bioactive compound is dissolved in oil droplets of emulsions that form in the gastrointestinal tract. Moreover, SOL is an environmentally responsive polymer, in which pH and temperature can influence the formation of micelles [31].

Therefore, the aim of this work was to develop SEDDS containing CO from *Copaifera reticulata* Ducke for per oral administration to improve the solubilization and consequent absorption of the CO’s bioactive substances in the gastrointestinal tract, thereby aiming to improve the treatment of leishmaniasis. Ternary phase diagrams were utilized during the design and preparation of the systems. The most stable formulations were selected after the physicochemical stability study, and these were submitted to emulsification testing using gastric and intestinal simulated media. The morphology of the selected systems was characterized by light microscopy and cryo-transmission electron microscopy. Moreover, their aqueous dispersions were also characterized in terms of their zeta potential, size, polydispersity index and drug dissolution. The in vitro activity was evaluated against the species *L. amazonensis* and *L. infantum*.

## 2. Materials and Methods

### 2.1. Materials

Polyethylene Glycol 400 (pure, pharma grade; PEG 400), sodium chloride, hydrochloric acid, acetic acid, sodium hydroxide and monobasic potassium phosphate were purchased from Synth (Sao Paulo, SP, Brazil). Acetonitrile (HPLC grade) was acquired from Honeywell Riedel-de-Haën (Morris Plains, NJ, USA). β-caryophyllene (CAR; standard with purity ≥ 80%) was purchased from Sigma-Aldrich (Saint Louis, MO, USA). Polyvinyl caprolactam-polyvinyl acetate-polyethylene glycol graft copolymer (Soluplus^®^; SOL) was kindly donated by BASF (Ludwigshafen am Rhein, Germany) and ultra-purified water was obtained in-house using a water purification system (Evoqua Water Technologies, Pittsburgh, PA, USA). The copaiba oil-resin (CO) from *Copaifera reticulata* Ducke was obtained from Copaíba da Amazônia company that sustainably obtains CO from rainforest trees at the agroextractive association Aripuana/Guariba (Apui, AM, Brazil). The collection and use of CO were authorized and follow the environmental legislation of the National System of Authorization and Information on Biodiversity (SISBIO n° 72922-1) and the National System of Genetic Heritage Management (SISGEN n° AE28797).

### 2.2. Construction of Ternary Phase Diagrams

Different compositions of CO, SOL, PEG 400 and/or ultra-purified water were screened on the basis of their maximum dispersibility [32]. Firstly, ternary phase diagrams were prepared for the development of emulsifying systems. The SEDDS diagram was composed of CO as the lipid phase, SOL as the surfactant and PEG 400 PEG400 (as a co-surfactant). The diagram was constructed by titration method using one of the SEDDS components and not water, as pseudo ternary diagrams are usually constructed [33]. Therefore, two SEDDS components (CO and SOL, CO and PEG 400 or SOL and PEG 400) were mixed at the ratios of 1:9, 2:8, 3:7, 4:6, 5:5, 6:4, 7:3, 8:2 and 9:1 (*w*/*w*), and the titration was performed with the third SEDDS component up to 33% (*w*/*w*) (Table S1). The EM diagram was composed of CO, SOL and ultra-purified water (Table S2) and constructed using the same method as for the SEDDS diagram, as previously described. The samples were mixed and kept at room temperature (25 ± 2 °C) until the equilibrium was reached for the addition of the next component. After each addition of a component, a visual inspection was performed. For SEDDS, the homogeneous samples without phase separation were evaluated according to their consistency and classified as liquid or viscous (+, ++ and ++++). In addition, other macroscopic characteristics were investigated. The milky or turbid preparations which did not display phase separation during the construction of diagrams were identified as emulsion systems (EM) and classified as liquid or viscous (+, ++ and +++).

### 2.3. Preparation of Formulations

For the preparation of 10.0 g of SEDDS formulations, 3.0 g of SOL was left in contact with 6.0 g of PEG 400. After 24 h, this binary mixture (SOL and PEG 400) was stirred manually at 45 ± 5 °C for 10 min, until complete SOL dispersion. Afterwards, 1.0 g of CO was gradually incorporated under manual agitation (100 µg added per minute) at 25 ± 2 °C.

Moreover, 10.0 g of EM formulations were prepared by dispersing gradually 3.0 g of SOL (0.5 g added per minute) in 6.0 g of ultra-purified water with constant manual stirring at 25 ± 2 °C. After complete SOL solubilization, 1.0 g of CO was added gradually (100 µg added per minute) under manual agitation, at 25 ± 2 °C, until complete homogenization. All the formulations were packaged in hermetically sealed containers and kept at room temperature prior to further analysis.

### 2.4. Preliminary Physicochemical Stability Study

The investigation of the preliminary physicochemical stability of the formulations was performed according to Cosmetics Stability Guide from the Brazilian Health Regulatory Agency (ANVISA) with some modifications [34]. Approximately 1 g of each formulation was submitted to centrifuging (3000 rpm; 946× *g*) for 30 min and at 25 ± 1 °C. Afterwards, another centrifuging process at 12,000 rpm (15,132× *g*) was performed for an additional 30 min at 25 ± 1 °C. The formulations that did not display phase separation after this last centrifuging step were submitted to freezing (−5 ± 2 °C; for 24 h) and thawing (40 ± 1 °C; for 24 h) cycles of 48 h each for 14 days. After the cycles of freezing and thawing, the formulations were submitted to another centrifuging at 3000 rpm (946× *g*) for 30 min and at 25 ± 1 °C. The formulations were evaluated regarding their organoleptic characteristics (color, aspect and smell), homogeneity, and phase separation.

### 2.5. Analysis of Emulsification Properties of Systems

#### 2.5.1. Preparation of Simulated Media

The gastric (GS) and intestinal (IS) simulated media (without enzymes) were prepared according to the Brazilian Pharmacopoeia [35]. The GS was prepared by dissolution of 2.0 g of sodium chloride in ultra-purified water, which was subsequently added to a 1000 mL volumetric flask with 7.0 mL of hydrochloric acid (concentrated), and the final volume was reached with the addition of ultra-purified water. The pH was adjusted to 1.2 ± 0.1 using hydrochloric acid or sodium hydroxide (1.0 mol/L). The IS was prepared by mixing 250 mL of 0.2 M monobasic potassium phosphate solution and 112 mL of 0.2 M sodium hydroxide solution in a 1000 mL volumetric flask. Afterwards, the final volume was reached with the addition of ultra-purified water. The pH was adjusted to 6.8 ± 0.1 using 0.2 M sodium hydroxide solution.

#### 2.5.2. Analysis of Emulsifying Properties

A 3 g sample of each formulation was submitted to titration with 50 µL aliquots of the different media (ultra-purified water, GS or IS) at 37 ± 2 °C. The macroscopic changes were observed (i.e., phase separation and variation in consistency), and pseudo-ternary phase diagrams were constructed.

#### 2.5.3. Determination of the Emulsification Time

The emulsification time was evaluated by adding 12 mg of each formulation to a test tube filled with 10 mL of medium (ultra-purified water, GS or IS). The analyses were performed at 37 ± 2 °C. The time required for the complete dispersion of the formulations to occur was determined by visual analysis. Afterwards, the formulations were homogenized and their absorbance was measured at the wavelength (λ) of 600 nm using a UV–Vis spectrophotometer model 1800 (Shimadzu, Tokyo, Japan) at room temperature [36,37,38,39]. For each formulation, the analysis was performed in at least three replicate samples.

### 2.6. Morphological Analysis of Selected Systems

#### 2.6.1. Analysis by Light Microscopy

The selected emulsifying systems were spread on a glass slide and analyzed under a light microscope (Kozo Optical and Electronic Instrument Company, Nanjing, China) with an original magnification of ×1000.

#### 2.6.2. Analysis by Cryogenic Transmission Electron Microscopy (Cryo-TEM)

The selected emulsifying systems were diluted at ratio 1:10 (formulation:water) in ultra-purified water to carry out the analyses. A 2 µL sample was applied to copper grids for electronic microscopy with Lancey carbon film, with the excess drying. The grids for cryomicroscopy were treated with a load of 25 mA for 50 s using EasiGlow equipment. Afterwards, the grids were led to the sample glazing robot. The sample grids were immediately frozen in liquid ethane and then kept in liquid nitrogen until analysis and acquisition of images in the electronic transmission microscope (JEM1400Plus, Tokyo, Japan).

### 2.7. Size Analysis and Zeta Potential 

The selected systems were diluted at a ratio of 1:10 (formulation:water) in ultra-purified water to carry out the analyses, and time was allowed for the systems to stabilize. The droplet diameter and the polydispersity index of the samples were determined using a droplet size analyzer (Particulate Systems, Norcross, GA, USA) using the dynamic light scattering technique at temperatures of 25 ± 0.5 °C and 37 ± 0.5 °C (room and body temperature, respectively). For determination of zeta potential, the systems were diluted at a ratio of 1:100 (formulation:water) in ultra-purified water. The zeta potential of the diluted formulations was determined using the electrophoretic mobility technique, at temperatures of 25 ± 0.5 °C and 37 ± 0.5 °C, in the same droplet size analyzer previously described. For all the formulations, the analyses were performed in at least three replicate samples.

### 2.8. In Vitro Dissolution Study

The in vitro dissolution analysis of CO from the selected formulations was performed using a previously validated high-performance liquid chromatographic (HPLC) method, using β-caryophyllene (CAR) as a marker [40]. The HPLC method consisted of the mobile phase composed of acetonitrile and ultra-purified water acidified with 0.1% (*v*/*v*) acetic acid. A gradient elution mode was utilized, under a flow rate of 1 mL/min and analysis time of 25 min. The HPLC device model Prominence-I LC-2030C 3D (Shimadzu, Tokyo, Japan), equipped to carry out automatic injections (20 µL), with an oven to control the temperature (22 °C) and a photodiode array UV-Vis detector, was utilized. The analyses were accomplished using an analytical column (Gemini C18, 4 × 3 mm; Phenomenex, Torrance, CA, USA) at a wavelength (λ) of 210 nm.

**Figure 1:**
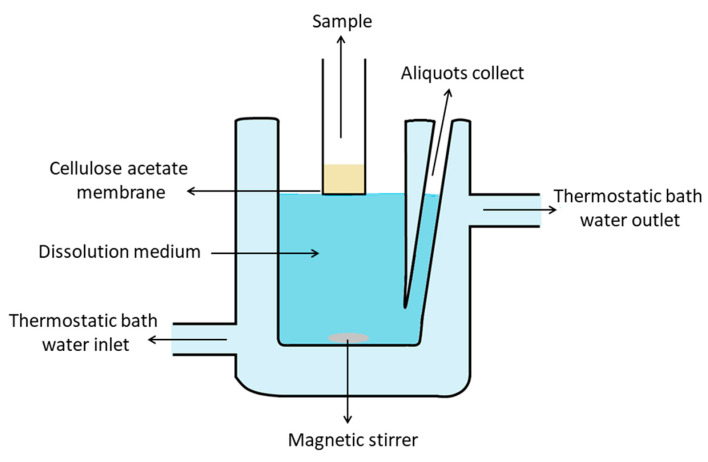
Graphical representation of vertical diffusion cell based on Franz’s model with modifications and used in the in vitro dissolution test.

The dissolution analysis was carried out in a vertical diffusion cell based on the Franz’s model with modifications (Figure 1). A 50 mL sample of dissolution medium (ultra-purified water, GS or IS) was utilized with controlled magnetic stirring at a temperature of 37 ± 0.5 °C using a thermostatic bath. Cellulose acetate membrane (molecular weight cut-off 12,400 Da; Sigma-Aldrich, Sao Paulo, SP, Brazil), previously hydrated in water, was used as support. About 120 mg of formulation was weighed and added along with 1 mL of dispersion medium onto the support membrane. Aliquots of 1.5 mL of the dissolution medium were collected at 15, 30, 60, 120, 240, 360 and 480 min, diluted in 2 mL of acetonitrile, and filtered through a membrane (0.45 µm opening, PTFE) for further determinations by the HPLC method. The analyses were carried out for at least three replicates of each sample. 

### 2.9. In Vitro Antiproliferative Activity

#### 2.9.1. Activity against Promastigote Forms

Promastigote forms of *L. amazonensis* (IFLA/BR/1967/PH8 transfected with pIR1SAT-LUC(a)DsRed2(b) (B5947)) and *L. infantum* (MHOM/MA/67/ITMAP-263 transfected with plasmid pSP72αHYGαLuc1.2) were grown in Warren medium (brain and heart infusion, hemin and folic acid; pH 7.2) supplemented with 10% inactivated fetal bovine serum (FBS) and incubated at 25 °C. Afterwards, the promastigote forms (1 × 10^6^ parasites/mL), after 48 h of culturing, were added to a sterile 96-well plate, in the presence and absence of the formulations, and then incubated for 72 h at 25 ± 1 °C. After the treatments, the cell viability assay was performed using the XTT reduction method [37]. This method is based on the ability of mitochondrial dehydrogenase enzymes to convert the colorless tetrazolium salt (2,3-bis-(2-methoxy-4-nitro-5-sulphenyl)-(2H)-tetrazolium-5-carboxanilide) into an orange substance derived from formazan. Thus, after the treatment period, XTT solution (0.5 mg/mL) was added for 4 h. The absorbance reading was performed at 450 nm in a plate spectrophotometer (Power Wave XS, BioTek, Winooski, VT, USA). The percentage of viable cells was calculated relative to the control (untreated). The inhibitory concentrations for 50% of the parasites (IC_50_) were determined by non-linear regression of the plotted values. Results were expressed as the mean ± standard deviation of at least three independent experiments.

#### 2.9.2. Activity against Amastigote Forms

To evaluate the antiproliferative activity against the amastigote forms of *L. amazonensis* or *L. infantum*, 5 × 10^5^ macrophages mL^−1^ were added, together with 5 × 10^6^ promastigotes mL^−1^ (ratio 1:10) in stationary growth phase, to white 96-well plates. They were incubated at 37 ± 1 °C, with 5% CO_2,_ during 24 h for internalization and differentiation of parasites into amastigotes forms.

The cells were then washed to remove non-internalized parasites and treated with increasing concentrations of the formulations and incubated for 48 h. Afterwards, the Pierce Firefly Luc One-Step Glow Assay kit (ThermoFicher, Rockford, IL, USA) was added according to the manufacturer’s instructions. Luminescence was quantified in a SpectraMax luminometer L (Molecular Devices, San Jose, CA, USA) with λ = 570 nm and an integration time of 1 s. The percentage inhibition of the parasites was calculated based on the luminescence of the control (untreated). The values were plotted, and the IC_50_ concentrations were calculated by non-linear regression [38]. The results are expressed as the mean ± standard deviation of at least three independent experiments.

### 2.10. Cytotoxicity Assay

J774A.1 macrophages were maintained in RPMI 1640 medium (Gibco; Grand Island, NY, USA), at pH 7.2, supplemented with L-glutamine and 10% FBS, and incubated at 37 °C in an atmosphere of 5% CO_2_. Cytotoxicity was evaluated in these macrophages using the cell viability assay through MTT reduction and considering the presence of CO [39,40]. This method is based on the ability of mitochondrial dehydrogenase enzymes to convert the water-soluble tetrazolium salt (3-(4,5-dimethylthiazol-2-yl)-2,5-diphenyltetrazolium bromide) into an insoluble purple substance called formazan. For this, J774A.1 macrophages (5 × 10^5^ cells/mL) in RPMI 1640 medium supplemented with 10% FBS were seeded in 96-well plates and kept at 37 °C and 5% CO_2_ for 24 h. Then, the formulations were added in increasing concentrations and the plate was incubated for 48 h. After treatment, the cells were washed with PBS (pH 7.2) and incubated in the presence of MTT (2 mg/mL) for 4 h. The supernatant was removed, the formazan crystals were solubilized in DMSO and the absorbance reading was performed at 570 nm in a plate spectrophotometer Power Wave XS (BioTek, Winooski, VT, USA). The percentage of viable cells was calculated in relation to the control. The CC_50_ (50% cytotoxic concentration for the cells) was determined by non-linear regression analysis. The results are expressed as the mean ± standard deviation of at least three independent experiments.

### 2.11. Statistical Analysis

The effect of a system’s composition on physicochemical characteristics was statistically evaluated by analysis of variance (ANOVA). In all cases, Tukey’s honestly significant difference post hoc test was utilized to perform comparisons of difference between sample means. Additionally, Student *t*-test was utilized for comparisons between two groups. In all cases, the level of significance of *p* < 0.05 was adopted. 

## 3. Results and Discussion

*C. reticulata* Ducke oil-resin is found in Copaifera trees, typically found in the Brazilian northeast and the Amazon regions. The exuded oil-resin is obtained by tapping the trunk of the tree. The CO utilized in this work was a transparent liquid, and the color varied from yellow to light brown. The process of obtaining CO, as well as the chemical composition, was standardized. The main constituents of CO are sesquiterpenes and diterpenes, with β-caryophyllene being the main sesquiterpene (around 40% of the total chemical composition), and copalic acid the main diterpene [41,42,43]. Therefore, the chemical compositions of copaiba oil-resins sourced from other geographical regions must be investigated to evaluate their quality and avoid high chemical variations and changes in order to confirm the results reported in this work.

### 3.1. Ternary Phase Diagrams

The ternary phase diagrams were obtained by titrations of the components (Tables S1 and S2), allowing the rapid investigation of a large number of samples in different compositions [44]. Despite SOL and PEG 400 forming a homogeneous mixture, we increased the temperature up to 45 °C in order to decrease the consistency, mainly of SOL, and facilitate mixing for all the ratios tested. Therefore, the evaluation of different proportions of each component in SEDDS and EM was possible.

**Figure 2:**
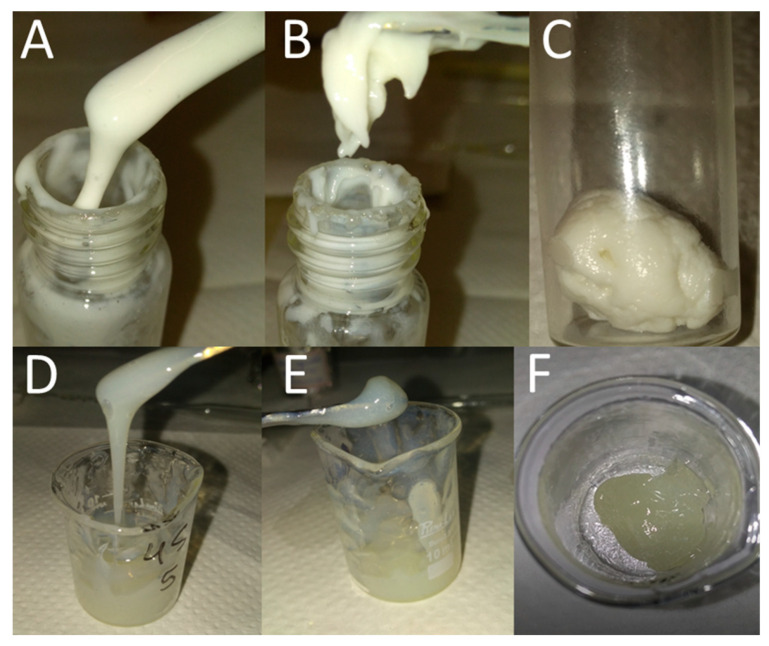
Macroscopic characteristics of systems. EM: (**A**) viscous +; (**B**) viscous ++; (**C**) viscous +++. SEDDS: (**D**) viscous +; (**E**) viscous ++; (**F**) viscous +++.

The process of emulsification of systems composed of CO, SOL and water was investigated (Figures S1–S8). Thus, the formulations were investigated by titration using one of the system components and constructing the ternary phase diagram. CO contains some components poorly soluble in the gastrointestinal environment, causing low bioavailability [20,21,22,23]. The development of an emulsion system containing SOL could improve the aqueous solubility of CO. Emulsions constitute a mixture of at least two liquids that are normally immiscible, stabilized by a surfactant, and most often of the oil-in-water type where the bioactive compound is dissolved in oil droplets of emulsions that form in the gastrointestinal tract [19]. The surfactant adsorption at the oil/water interface causes the lowering of the interfacial tension, decreasing the droplet size and assisting the dispersion [32,45]. In this way, EM systems were formed with different amounts of oil, water and surfactant; and the proportions between them are one of the important factors to obtaining stable formulations. Moreover, they can be utilized for comparisons with SEDDS using CO and SOL + PEG 400 to obtain improved systems (Figure 2).

In this work, to obtain the systems containing CO, a study of the mixture behavior with different proportions of components was required [44,46]. A ternary phase diagram was obtained by titration using the components of the systems (Figure S1). The formulations obtained in this study were classified according to their macroscopic characteristics. The different consistency aspects were evaluated by visual analysis and are represented by crosses (+), according to Figure 2A–C. It can be seen that all the formulations showed a whitish color, and consistency was the main characteristic that differentiated them (Tables S3–S5). Thus, the classification of consistency difference was performed by visual analysis and is represented by crosses (+), according to Figures S2–S4.

**Figure 3:**
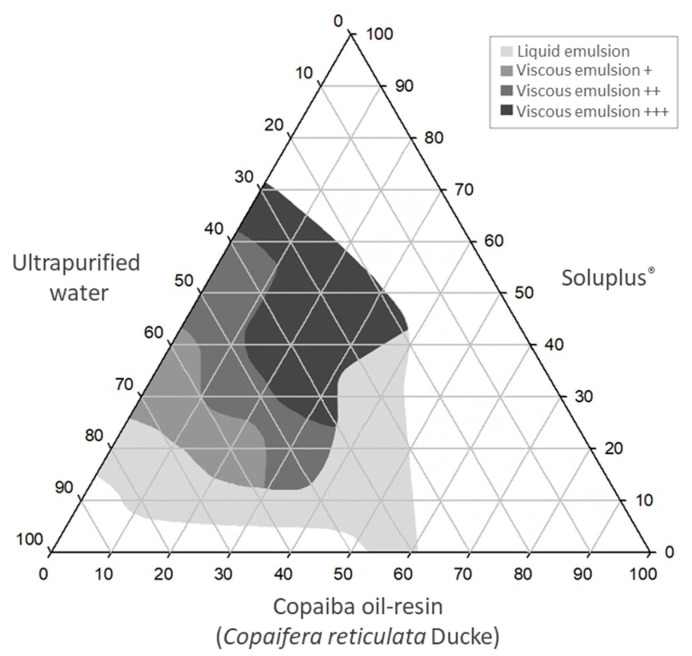
Ternary phase diagram of the emulsion systems (EM).

Therefore, the EM ternary phase diagram was constructed (Figure 3). The areas delimited in gray scale represent the EM obtained, and the white areas represent the phase separation. The EM containing OC could be obtained using up to 60% of OC, up to 70% of SOL and around 30 to 85% of water.

Based on the understanding the emulsifying behavior of CO plus SOL in an aqueous environment, the ternary phase diagram for SEDDS was constructed using CO, SOL and PEG 400. SEDDS are usually utilized as a technological strategy for oral administration of drugs belonging to Class II (low solubility and high permeability), according to the Biopharmaceutical Classification System [47], and CO probably belongs to this class. These systems consist of a mixture of oils and surfactants that form an emulsion in the gastrointestinal tract by contact with the aqueous medium and brand agitation [48]. 

During the development of SEDDS, the SOL was dispersed in PEG 400. SOL is a copolymer that has PEG 6000 as a structural component. Therefore, the addition of PEG 400 to the system was carried out with the objective of facilitate the SOL dispersion. Furthermore, PEG 400 is frequently used as a co-surfactant to aid in the emulsification process in the gastrointestinal tract in self-emulsifying systems [26,49]. Therefore, the SEDDS formulations were composed of three components: CO as the oil phase, SOL as the surfactant and PEG 400 as a co-surfactant. The ternary phase diagram was utilized to identify the optimal proportions among these components. The SEDDS diagram regions explored are displayed in Figure S5.

**Figure 4:**
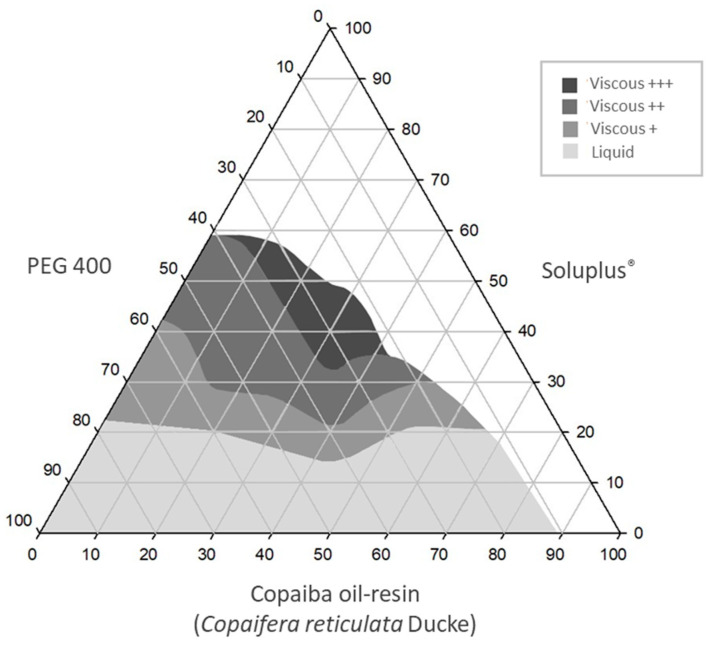
Ternary phase diagram of self-emulsifying systems (SEDDS).

For the diagram development (Figures S6–S8), the SEDDS were classified according to formulation consistency by visual analysis (Tables S6–S8), which is represented by crosses (+), as seen in Figure 2D–F. Moreover, unlike the EM, the SEDDS showed a more yellowish or pearly color, depending on the amount of CO added. Figure 4 displays the SEDDS ternary phase diagram. The areas delimited in gray scale represent the SEDDS obtained, and the white areas represent separation of phases.

### 3.2. Preparation and Preliminary Physicochemical Stability Study

Emulsion systems are based on the dispersion of an immiscible liquid (droplets) in a continuous phase [45,50]. SEDDS are dispersions of oils and surfactants that can emulsify on contact with the aqueous environment of the gastrointestinal system, as reported in [19,26], for example. In view of these considerations, due to both systems (EM and SEDDS) being dispersions, the droplets are influenced by gravity, which can generate instability phenomena in the formulations [32,45].

The formulations selected for the analysis by the centrifuging test were chosen according to their presence in the emulsification region of the EM diagram, and the region of homogeneity of the SEDDS diagram (Figure S9). Thus, a total of 15 EM and 21 SEDDS were submitted to the centrifuging tests. Table S9 displays the composition of these formulations.

The stability is considered suitable when there is no change in appearance, odor or other physical properties [45]. Therefore, the visual inspection of phase separation over time was considered as indicative of instability [50], and this was determined by visual inspection of the formulations. Some important differences between the EM and the SEDDS were observed. Among the 15 EM formulations, only three (E1, E2 and E3) did not show phase separation at 3000 rpm (946× *g*) and only one (E1) at 12,000 rpm (15,132× *g*). However, from the 21 SEDDS, 16 (F4, F5, F6, F7, F9, F10, F12, F13, F14, F15, F16, F17, F18, F19, F20 and F21) did not show phase separation at 3000 rpm (946× *g*), and 10 (F6, F7, F10, F13, F15, F16, F17, F18, F20 and F21) did not at 12,000 rpm (15,132× *g*). This fact corroborates that the SEDDS are commercially developed to reduce the instability present in emulsions [19]. In addition, most of the formulations that did not show phase separation after the centrifuging test were the ones that had the highest consistency. This is explained by the fact that rising or settling of droplets can be slowed down by an increase in the formulation viscosity [50].

Afterwards, the formulations selected by the centrifuging test at 3000 rpm or 946× *g* (E1, E2, E3, F4, F5, F6, F7, F9, F10, F12, F13, F14, F15, F16, F17, F18, F19, F20 and F21) were submitted to a preliminary physicochemical stability study. The formulations that showed phase separation at 12,000 were also included because they were selected according to ANVISA guide [34].

The preliminary physicochemical stability study involves the use of extreme temperature conditions to accelerate possible instabilities, so the formulations were submitted to cycles of freezing (−5 °C) and thawing (40 °C) [34]. After these cycles, only one EM (E1) and nine SEDDS (F4, F5, F6, F7, F12, F13, F14, F17 and F18) did not show phase separation. 

After the freeze–thaw cycles, these formulations were also submitted to centrifuging at 3000 rpm (946× *g*) for 30 min. In this test, one EM (E1) and six SEDDS (F7, F12, F13, F14, F17 and F18) did not display phase separation. Therefore, these formulations were selected for the further tests.

### 3.3. Analysis of Emulsification Properties of Systems

The current leishmaniasis treatment is generally based on the parenteral administration route of drugs, with miltefosine being the only drug administered orally; however, it has displayed teratogenicity and is expensive [3,51]. Thus, the development of new formulations for leishmaniasis treatment with a more comfortable and safer route of administration, like the oral route, is necessary.

When developing a formulation for oral administration, some physiological conditions of the gastrointestinal tract which can influence drug absorption must be considered. Among these conditions, the water solubility is the more important because compounds that are poorly water soluble have low dissolution in gastrointestinal fluids [19]. Thus, it is important to investigate how the selected systems (EM and SEDDS) behave when in contact with the gastrointestinal fluids. 

**Figure 5:**
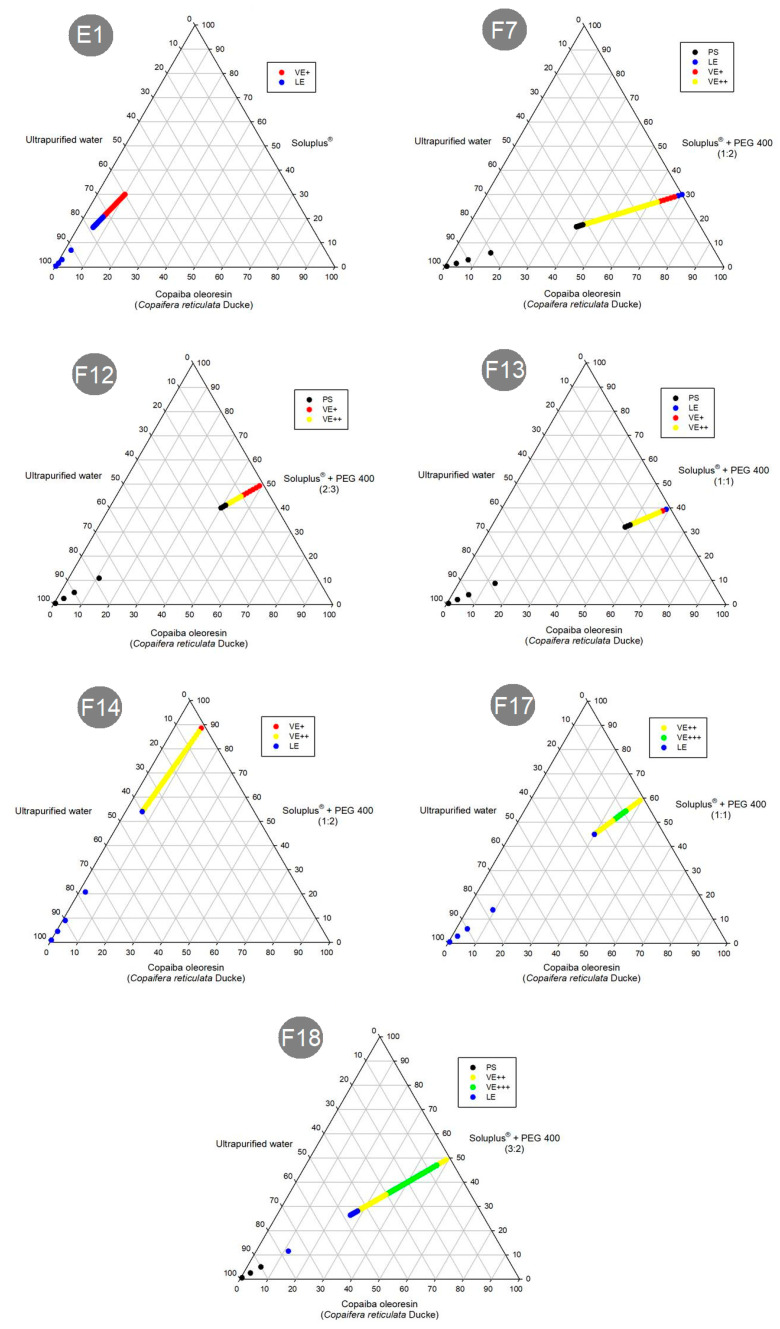
Schematic diagrams of the tests and results of emulsification analysis of formulations in ultra-pure water: PS (phase separation); VE (viscous emulsion); LE (liquid emulsion).

**Figure 6:**
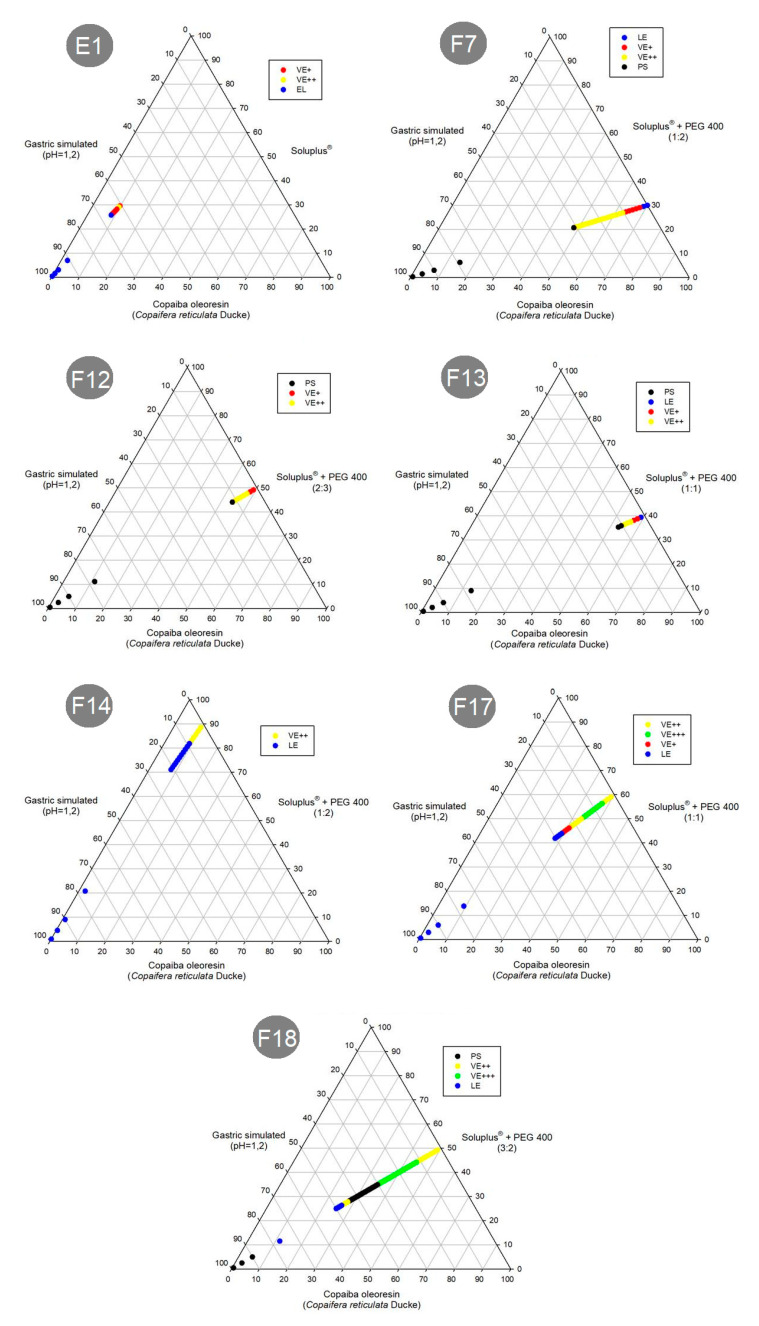
Schematic diagrams of the tests and results of emulsification analysis of formulations in gastric simulated (GS) media. PS (phase separation); VE (viscous emulsion); LE (liquid emulsion).

**Figure 7:**
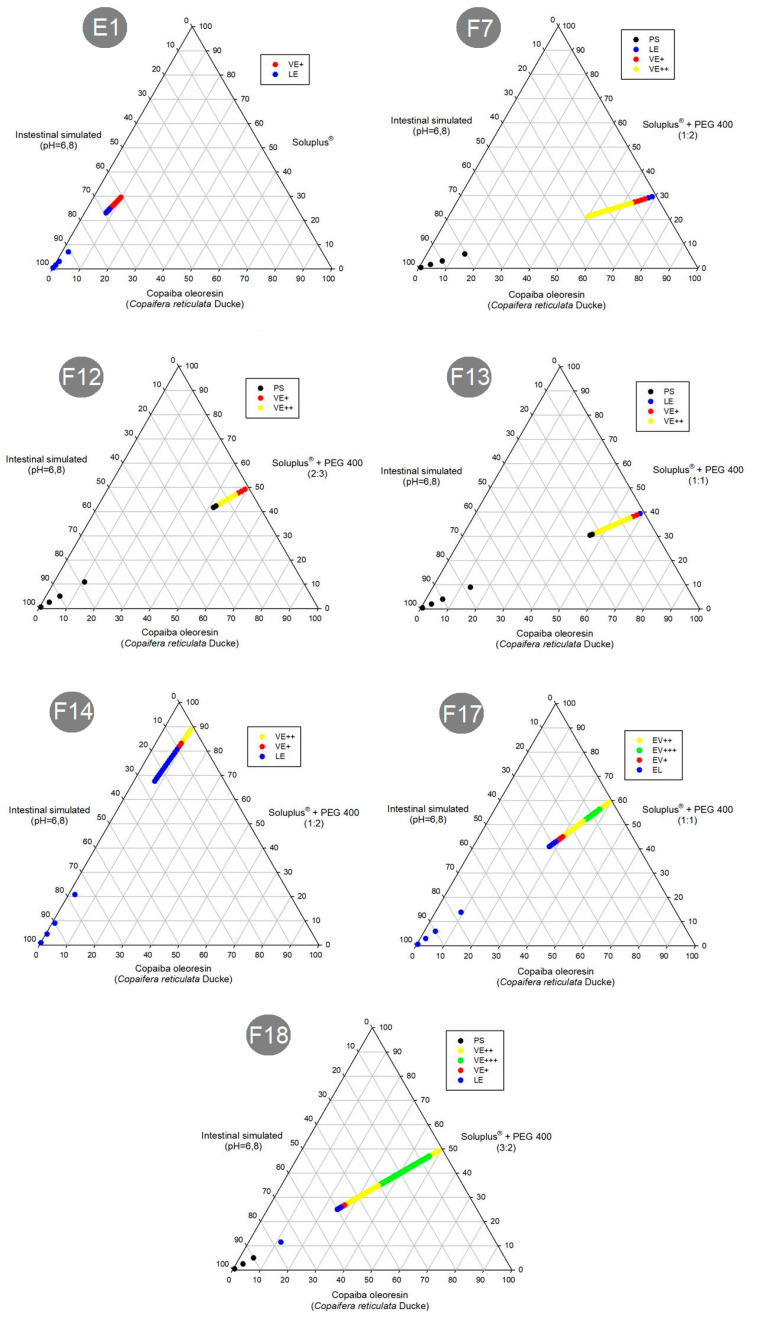
Schematic diagrams of the tests and results of emulsification analysis of formulations in intestinal simulated (IS) media: PS (phase separation); VE (viscous emulsion); LE (liquid emulsion).

In this work, an in vitro preliminary investigation was carried out by subjecting the selected formulations to agitation with ultra-purified water, IS or GS, at body temperature (37 °C) (Figure 5, Figure 6 and Figure 7).

The diagrams above indicate that the formulations F7, F12, F13 and F18 entered a separation phase in the mixtures. However, formulations E1, F14 and F17 were emulsified in any media proportion. Therefore, only the formulations with the low amounts of CO and greater amounts of SOL (E1, F14 and F17) could emulsify with any proportion of media.

**Figure 8:**
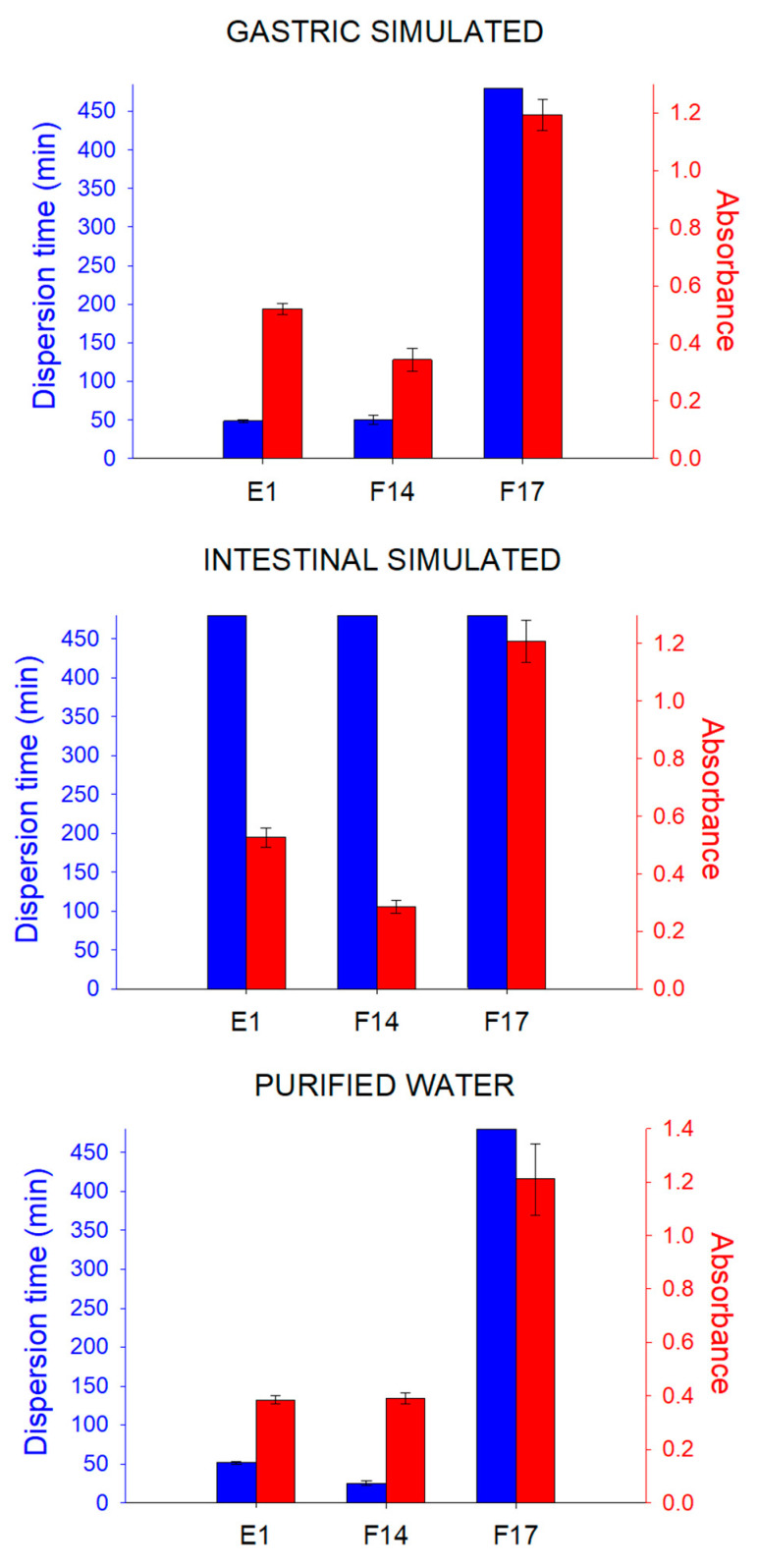
Dispersion time (blue) and absorbance (red) values of formulations dispersed in three different media: gastric simulated (GS); intestinal simulated (IS); purified water.

In addition, to investigating the emulsification process of these formulations, the time required for dispersion was also investigated. Figure 8 shows the dispersion time of E1, F14 and F17. However, the SEDDS F7, F12, F13 and F18 did not display complete dispersion after 8 h (480 min) in the three dispersion media (purified water, IS and GS). This was a very long time, which may be due to the characteristics of SOL. Comparing the composition of these formulations and the dispersion media with the diagrams of the emulsification tests (Figure 5, Figure 6 and Figure 7), it was possible to determine that phase separation of the formulations F7, F12, F13 and F18 occurred, and therefore, they do not have a dispersion time. 

The formulation F17 did not show complete dispersion in the three dispersion media after 8 h. However, in contrast to the previously mentioned results for the other SEDDS formulations (F7, F12, F13 and F18), the diagrams obtained in the emulsification test (Figure 5, Figure 6 and Figure 7) demonstrate that F17 has a dispersion capacity in this proportion in the media, with no phase separation. Thus, the formulation F17 has a dispersion time greater than 8 h.

E1 demonstrated a mean dispersion time of 48.67 and 51.67 min in purified water and GS, respectively, with no significant difference (*p* = 0.148) between them. However, the dispersion time in IS was greater than 8 h. Thus, it can be inferred that with a pH increase from purified water to IS, there was an increase in E1 dispersion time. Formulation F14 displayed a mean dispersion time of 25.33 and 50.00 min in purified water and GS, respectively, with a significant difference (*p* = 0.007) between them, while in the IS, the dispersion time was greater than 8 h. It is probable that this very long dispersion time was due to SOL preventing a rapid solubilization of the CO components.

This pH influence on dispersion time probably is due to the SOL. Recent studies have shown that environmental conditions, such as temperature and pH, can influence the formation of SOL micelles. Moreover, the increase in temperature until reaching a critical temperature (T_sol/gel_), causes a transition of the SOL liquid-like solution into an elastic gel structure (sol/gel transition) [52]. With the increase in temperature, the PEG 6000 chains of SOL, located outside the micelles, can dehydrate due to the breaking of the hydrogen bonds between water and these PEG chains, causing aggregation of the micelles due to a greater interaction between them until a state of gelation is reached, which is a reversible process with decreasing temperature [31,52].

Other parameters that can influence the T_sol/gel_ of SOL are the pH and presence of salts. Alopaeus and colleagues [31] determined the T_sol/gel_ of SOL in different media. It was observed that the T_sol/gel_ in 0.1 M hydrochloric acid (pH = 1.2) was approximately 38 °C, in ultra-purified water it was 36 °C and in phosphate-buffered saline (PBS, pH = 7.4) it was 33 °C. This media pH influence on the T_sol/gel_ was also demonstrated by Wu and collaborators [52], where they showed that the T_sol/gel_ of SOL in PBS pH = 5 and in PBS pH = 7.4 was 35.8 °C and 33.9 °C, respectively. Thus, these previous studies show that the pH value, with the presence of salts and the ionic strength of the medium, influences the T_sol/gel_ of the SOL and as the pH increases, the T_sol/gel_ of the system decreases. This can be explained by the presence of salts that can strongly interact with water molecules in the medium, making the polymer segments more likely to be excluded from water solvation, thereby favoring hydrophobic interactions between the polymer segments. Considering the dispersion time of formulations F14 and E1, with the increase the pH medium, the dispersion time increased, so it is probable, as in the studies mentioned above, that the formulations in IS at 37 °C have a gel-like structure, which results in greater difficulty for water to enter the system and thereby increases the dispersion time.

Moreover, the time that a pharmaceutical form takes to transit through the stomach depends on the pharmaceutical system, whether the organ is full (fed) or empty (fasting), and also the individual physiological conditions. Generally, this time can vary from 5 min to 2 h, although times of up to 12 h have been reported [19].

When fasting, the electrical activity of the stomach (interdigestive myoelectric complex) controls its motility, causing the transit of pharmaceutical forms through the organ. Stomach motility in this unfed condition comprises a four-phase cycle. Phase one is characterized by a period of inactivity with rare contractions for 40 to 60 min. Phase two is relatively similar to phase one, with the presence of more contractions. Phase three is when the strongest contractions occur, causing the opening of the pyloric sphincter, allowing removal of part of the stomach contents. Finally, phase four is a short transition period between the strong contractions of phase three and the inactivity of phase one [19]. Analyzing the dispersion time of formulations in the GS, it is possible to realize that the formulations F14 and E1 probably disperse faster than the F17 during the stomach transit.

The small intestine is considered the main site of absorption. Therefore, the transit time of pharmaceutical dosage forms through this organ is important for the drug’s bioavailability. The intestine movements are propulsive and mixing and normally have a transit time of 3 to 4 h, although slower and faster times have been reported [19]. Considering the dispersion time of the formulations in the IS, it could be concluded that their dispersion times are longer than the intestinal transit [19]. However, considering the passage of the pharmaceutical form through the stomach before the small intestine and that the formulations demonstrated a faster dispersion time at lower pH, it can be concluded that the formulations may be almost completely dispersed, or with their structure mostly dispersed, in the small intestine, therefore allowing a more prolonged dispersion in the intestinal lumen.

After determining the dispersion time, the formulations that displayed complete dispersion in the tested media proportions had their absorbance determined. The absorbance was measured at a wavelength in the visible spectrum (λ = 600 nm) to determine the turbidity existing after dispersion. Only formulations F14, F17 and E1 could be dispersed in the media proportions tested (Figure 8). The turbidity of a system may be related to the size of the colloidal material involved [19]. Formulation F17 appeared to have larger micelles than F14 and E1. Moreover, the self-emulsifying system F14 displayed a lower absorbance compared with E1, indicating that F14 possibly had micelles of a smaller size. The reason could be the presence of the co-surfactant PEG 400 in the F14 composition. Despite the simplistic and preliminary in vitro test, the results indicate that SOL is unsuitable for most of the formulations due to the long emulsion formation time. Therefore, formulations F14 and E1 were selected for the further analysis. 

### 3.4. Morphological Analysis

The size analysis of the droplets in formulations is important for predicting stability and absorption. Emulsions with larger droplets are more likely to coalesce, and those that show droplets with smaller diameters tend to be more stable. Furthermore, with respect to oral administration, the absorption of an emulsion improves as the droplets decrease in size [19]. The morphological analysis of the selected formulations was carried out using light microscopy and Cryo-TEM, in addition to size analysis by dynamic light scattering.

**Figure 9:**
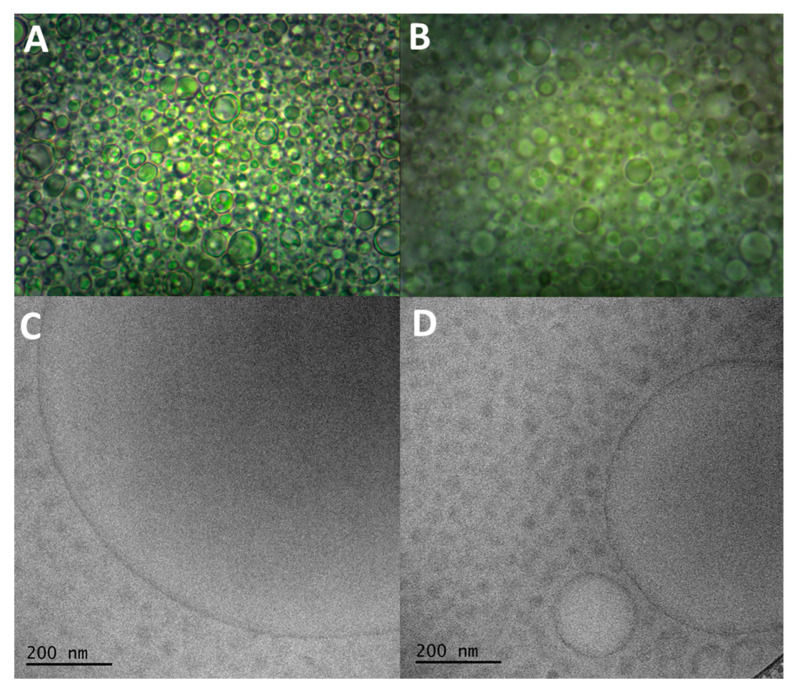
Morphological characteristics of emulsion system E1 (**A**,**C**) and self-emulsifying system F14 (**B**,**D**). Images obtained by light microscopy (**A**,**B**) and cryogenic transmission electron microscopy (**C**,**D**).

The light microscopy analysis of the formulations was carried out to visualize their structure (Figure 9A,B). Both formulations (E1 and F14) displayed structures similar to droplets. The SEDDS are characterized when the emulsification process occurs under gentle agitation, after coming into contact with the aqueous medium [19,26]. However, formulation F14 presented droplets similar to those of E1 (Figure 9B), even without the addition of water. This can be explained by the fact that PEG 400 is polar [53] and, therefore, acts like an aqueous phase. 

During the dynamic light scattering analysis, a beam of light is focused on the dispersed system and the equipment detects the light scattering that has occurred due to the presence of the droplets [54]. The size analysis was performed at temperatures of 25 and 37 °C for both systems (F14 and E1), and the systems were diluted at a ratio of 1:10 (formulation:ultra-purified water). Table 1 displays the mean diameter and polydispersity index of each formulation, while the Figure S10 shows the droplet size distribution.

At 37 °C, system E1 showed a mean diameter of droplets smaller than at 25 °C. It is probable that the temperature increase contributes to a better structuring of the system. It is also probable that with the increase in temperature, there is a reduction in the thermodynamic tendency, reducing the interfacial tension and causing a decrease in the size of the droplets [32,45]. This behavior was not observed for F14 which displayed mean diameter increases as the temperature rose. F14 contains PEG 400 as a co-surfactant, which contributes to the emulsifying process and reduces the interfacial tension and the size of droplets. This was observed in relation to E1 at 25 °C. However, as the temperature increased to 37 °C the droplet size of F14 also increased. The thermoresponsiveness of SOL may be involved in this behavior of the systems. As the temperature increases, the molecules of SOL change their spatial conformation, exposing more their hydrophilic regions and could increase their droplet size [29,30,31]. This behavior was also evidenced by the polydispersity index, since at 25 °C, formulation E1 showed a higher value and a bimodal distribution (Figure S10). According to the literature, the so-called nanostructured systems generally refer to formulations with droplet diameters below 200 nm, although there may be differences in classifications [19]. Thus, it can be inferred that formulation F14 is a nanostructured system at both temperatures (25 and 37 °C). However, E1 displayed droplets of nanometric size only at body temperature (37 °C). As previously mentioned, the absorption of an active agent improves with decreasing diameter of the droplets [19]. Therefore, formulations E1 and F14 would probably have a good absorption rate, due to their droplets being in the nanometer scale at 37 °C.

The nanometric sizes found in the formulations by dynamic light scattering were confirmed by transmission Cryo-TEM (Figure 9C,D). It was determined that both formulations (E1 and F14) had droplets mostly in sizes smaller than 200 nm. In addition, it was possible to visualize that the droplets had a spherical shape and a considerably uniform distribution. Moreover, SEDDS F14 displayed a lower tendency for droplets coalescence, due to their small size at room temperature (25 °C), indicating greater stability than for E1. 

The zeta potential is defined as the potential difference present between the surface of a droplet/particle and the electrically neutral region in the scattering. The magnitude and/or absence of these charges are important for the stability of dispersed systems, such as emulsions. Thus, a certain charge on the surface of droplets/particles originates repulsion between them that prevents sedimentation and the encounter of one droplet/particle with another [45]. During the development of dispersed systems, the zeta potential is commonly analyzed to determine possible instabilities in the formulations.

Both formulations (E1 and F14) showed negatively charged droplets at 25 °C and 37 °C (Table 1). Dispersed systems containing SOL display negative charges on the surface of droplets due to the dissociation of the hydroxyls present in the polymeric chains which occurs in the aqueous medium [31]. The systems displayed the highest zeta potential at 37 °C, with values of −14.68 and −18.05 mV for E1 and F14, respectively. Thus, both formulations displayed greater stability at 37 °C.

### 3.5. Dissolution Study

**Figure 10:**
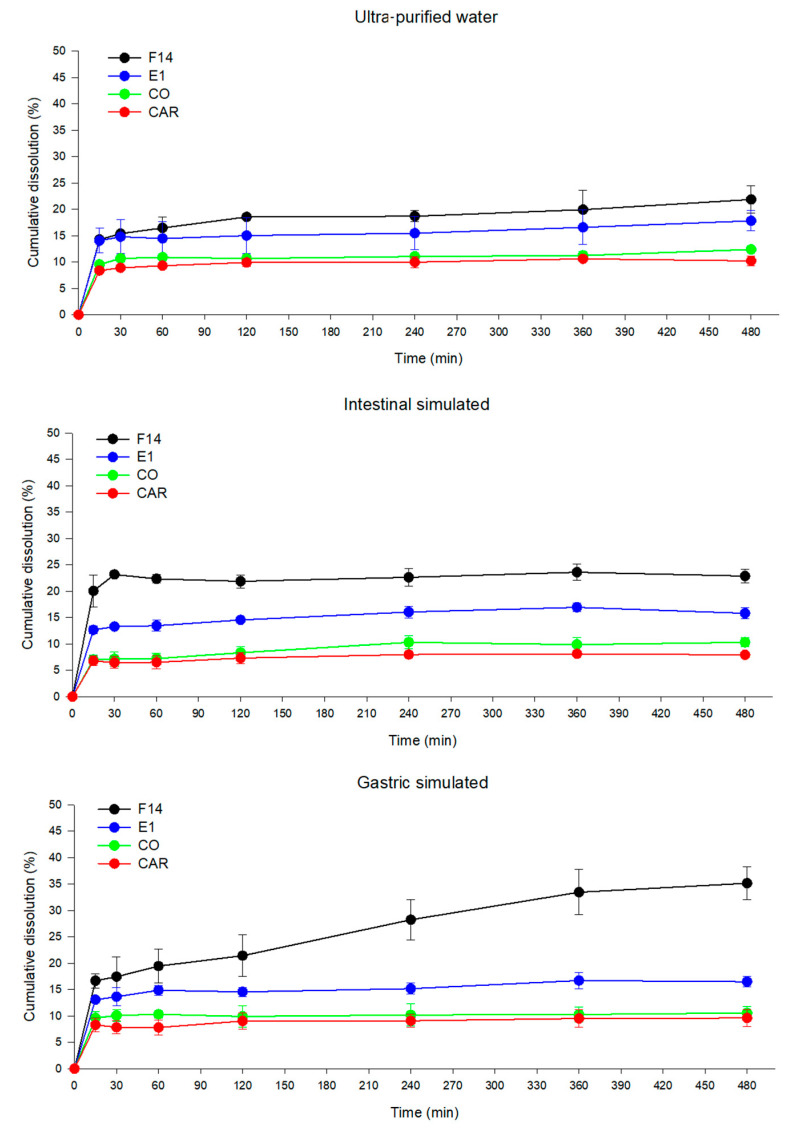
Dissolution profile of the emulsifying systems F14 and E1, the copaiba oil (CO) and the β-caryophyllene standard (CAR), using ultra-purified water, gastrointestinal simulated (GS) and intestinal simulated (IS) as the dissolution medium. Results are expressed as mean ± standard deviation (error bars) of three experiments (*n* = 3) carried out independently.

The applied technological strategy and some environmental factors can cause quantitative and qualitative changes in in vivo drug solubility. Therefore, in vitro dissolution studies may help to estimate the biological performance of the bioactive agent in vivo and is considered relevant to studying formulations with controlled release [55]. The CO dissolution profiles for the systems, based on CAR as a marker, are displayed in Figure 10.

Formulation E1 displayed a cumulative dissolution percentage of around 16% up to 8 h in all the dissolution media (GS, IS and ultra-purified water), indicating a prolonged dissolution process of the marker. Comparing the dissolution profiles of E1 in the three dissolution media, there was no significant difference (*p* > 0.05) between them. It was also observed that E1 released 1.44, 1.56 and 1.53 times greater cumulative amounts of CAR in water, GS and SI, respectively, compared with CO. Thus, E1 increased the CO solubility in the dissolution media, thus reflecting an increased CAR release.

Formulation F14 displayed a significant difference (*p* < 0.05) in levels of CAR cumulative dissolution in the different media. There was a higher percentage of CAR solubilized in GS than in the other dissolution media (Figure 10). Furthermore, formulation F14 had 1.77, 3.33 and 2.22 times greater CAR dissolution compared with CO in the ultra-pure water, GS and IS, respectively. This result indicates that formulation F14 improved the CO solubility in all tested dissolution media. 

By comparing the dissolution profiles of E1 and F14, it was possible to observe that F14 achieved higher percentages of cumulative CAR dissolution in the three media. This can be explained by the presence of PEG 400, which can favor the process of emulsification, resulting in the improvement in the release/dissolution of CO. However, this methodology showed the CO was still little solubilized in dissolution media close to the in vivo conditions. The dissolution rate was not highly increased in the formulation. The cellulose acetate membrane used as a sample support could interact with the micelles of system and impair their passing to the receptor medium. Further in vivo investigations are necessary to show the ability of systems in solubilizing CO. This response could be investigated by, for example, performing pharmacokinetic and bioavailability studies of CO after oral administration of the developed systems.

### 3.6. Anti-Leishmanial Activity and Cytotoxic Evaluation

It has already been demonstrated that CO has anti-Leishmania activity [14,56]. Thus, the in vitro antiproliferative activity of CO and the formulations was investigated against the promastigote and amastigote forms of the protozoa of *L. infantum* and *L. amazonensis* that can cause visceral and cutaneous leishmaniasis, respectively [55]. The promastigote form is found in the insect’s saliva and is transmitted to the host’s bloodstream during the bite, while the amastigote is the form which multiplies within the cells of the host’s monocyte phagocytic system [57,58,59].

Table 2 shows the concentrations achieving 50% inhibition of protozoa (IC_50_) for the promastigote and amastigote forms of *L. amazonensis* and *L. infantum.* The results are expressed in relation to the total formulation, while the results shown in parentheses are in relation to the CO concentration. 

The CO IC_50_ values against promastigote forms were slightly higher than those previously reported. Saintos and collaborators [56] found IC_50_ values for the species *C. reticulata* Ducke against promastigote forms of *L. amazonensis* of around 22 and 5 µg/mL, whereas the activity against intracellular amastigote forms was in accordance with previously reported values of in vitro antiproliferative activity of around 20 µg/mL [14].

In addition, the CO did not demonstrate significant differences in antiproliferative activity (*p* = 0.3310) against *L. amazonensis* and *L. infantum* species in the promastigote form. However, a significant difference (*p* = 0.0143) was observed in relation to the amastigote form. Furthermore, both species showed a significant difference (*p* < 0.05) regarding the antiproliferative activity against their amastigote and promastigote forms. Thus, it was demonstrated that the CO used in this study showed better results in relation to amastigote forms, mainly of the *L. infantum* species. 

Considering the formulations, all the samples showed a certain level of antiproliferative activity. Furthermore, they showed significant higher activity (*p* < 0.05) against amastigote than promastigote forms in both species (*L. amazonensis* and *L. infantum*). In addition, the other constituents of systems (SOL and PEG 400) apparently do not show activity against the parasites, since the samples without the CO (BE1, BF14) showed very high IC_50_ values.

**Figure 11:**
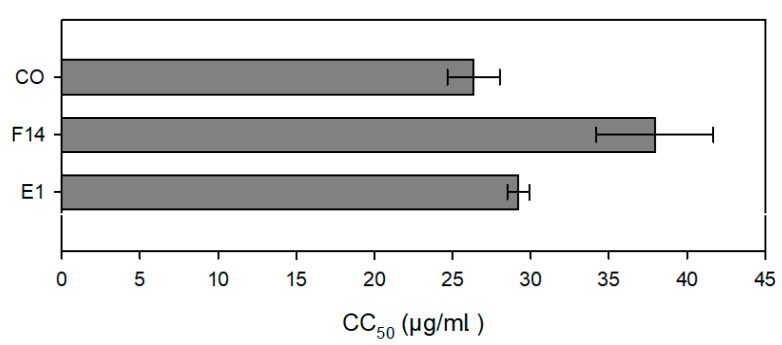
Cytotoxicity of selected formulations (F14 and E1) and CO (CC_50_) against macrophages.

Table 2 and Figure 11 also display the in vitro cytotoxicity of CO and the formulations against macrophage cells. The formulations and CO all displayed low cytotoxic concentrations, as indicated by the CC_50_ values (concentrations that toxic to 50% of the cells). Moreover, formulation F14 was the least cytotoxic. 

**Figure 12:**
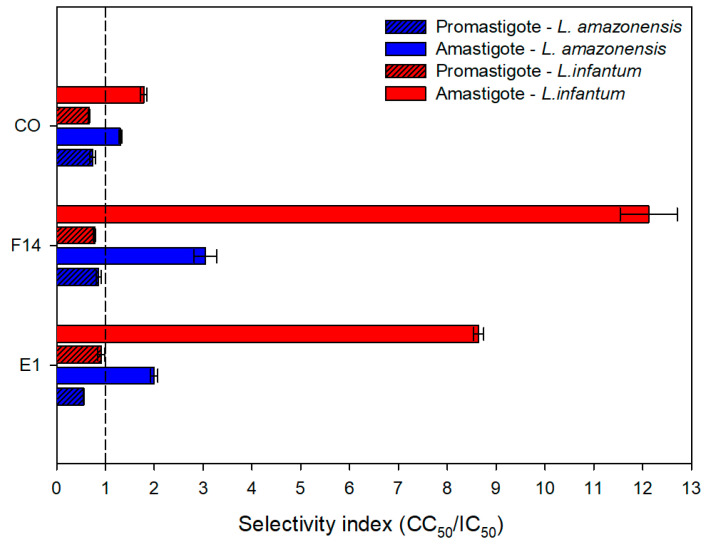
Selectivity index of selected formulations (F14 and E1) and copaiba oil-resin (CO) against amastigote and promastigote forms of *L. amazonensis* and *L. infantum*.

The cytotoxicity of the samples was compared with the antiproliferative activity, resulting in the selectivity index (Figure 12). Values greater than one on the selectivity index (SI) are considered more selective for parasites [56]. It was observed that the formulations, and the CO showed SI greater than one in relation to amastigote forms, especially against *L. infantum*.

After the oral absorption of systems, the drug release occurs and the bioavailable drug is exposed to the parasites. This in vitro test shows some limitations that do not reflect the in vivo activity. However, this analysis helped to investigate the performance of formulations, considering their previously determined physicochemical characteristics. Therefore, formulations E1 and F14 displayed the best dispersion ability in the media (GS, IS and purified water). Moreover, these formulations had low concentrations of CO, low cytotoxicity and greater effects.

## 4. Conclusions

Emulsifying systems developed using SOL as a surfactant proved to be a good technological strategy for use in the treatment of leishmaniasis. According to the emulsification test, and antiproliferative activity and cytotoxicity tests, the most suitable formulations were system E1 (10% CO; 30% SOL; 60% ultra-purified water) and the self-emulsifying system F14 (10% CO; 30% SOL; 60% PEG 400). The physicochemical characterization of these selected systems was carried out, and the results were assessed according to their applicability for oral administration. Furthermore, the developed formulations were shown to be environmentally responsive and nanostructured emulsion systems. SEDDS F14 and the emulsion E1 have potential applicability in the treatment of leishmaniasis by oral administration. SEDDS F14 is a candidate for further pharmacokinetics studies as well as in vivo investigations against leishmaniasis. However, further studies are necessary to evaluate the effectiveness of SOL in this system.

## Figures and Tables

**Table 1:** Mean diameter, polydispersity index and zeta potential of formulations E1 and F14 (aqueous dispersions) at different temperatures (25 and 37 °C). The results are the mean (±standard deviation) of at least three replicate samples for each formulation.

Formulations	Mean Diameter (nm)		Polydispersity Index		Zeta Potential (mV)	
	25 °C	37 °C	25 °C	37 °C	25 °C	37 °C
E1	716.07 ± 52.94	81.27 ± 4.19	0.50 ± 0.01	0.31 ± 0.01	−5.85 ± 0.20	−14.68 ± 0.12
F14	76.50 ± 0.30	122.63 ± 2.05	0.38 ± 0.01	0.23 ± 0.02	−12.82 ± 0.79	−18.05 ± 2.52

**Table 2:** Cytotoxicity and antiproliferative activity of selected formulations (F14 and E1) and copaiba oil-resin (CO) against promastigote and amastigote forms of *L. amazonensis* and *L. infantum*.

Sample	CC_50_ (µg/mL)	*L. amazonensis*		*L. infantum*	
		Promastigote	Amastigote	Promastigote	Amastigote
		IC_50_ (µg/mL)	IC_50_ (µg/mL)	IC_50_ (µg/mL)	IC_50_ (µg/mL)
CO	26.34 ± 1.68	35.89 ± 4.98	20.20 ± 1.70	39.43 ± 1.81	14.80 ± 1.48
E1	292.06 ± 7.10 (29.21 ± 0.71)	525.77 ± 19.25 (52.58 ± 1.93)	146.50 ± 9.06 (14.65 ± 0.91)	320.47 ± 32.02 (32.05 ± 3.20)	33.81 ± 1.24 (3.38 ± 0.12)
BE1 **	>2000	>2000	>1000	>2000	>1000
F14	379.38 ± 37.39 (37.94 ± 3.74)	441.24 ± 17.51 (44.12 ± 1.75)	124.34 ± 2.77 (12.43 ± 0.28)	488.13 ± 37.72 (48.81 ± 3.72)	31.24 ± 1.58 (3.12 ± 0.16)
BF14 **	>1000	>2000	>1000	>2000	>1000

Control (no treatment) > 2000 (for both Leishmania species and forms); ** mixture of formulation compounds without CO.

## Data Availability

The data that support the findings of this study are available from the corresponding author upon reasonable request.

## Supplementary Materials

The following supporting information can be downloaded at: https://www.mdpi.com/article/10.3390/pharmaceutics15082127/s1. Table S1. Scheme of titrations performed to obtain the ternary phase diagram of self-emulsifying drug delivery systems (SEDDS) composed of copaiba oil-resin (CO), Soluplus (SOL) and polyethylene glycol 400 (PEG 400). Table S2. Scheme of titrations performed to obtain the ternary phase diagram of emulsion systems (EM) composed of copaiba oil-resin (CO), Soluplus (SOL) and ultra-pure water. Figure S1. Ternary phase diagrams of emulsion systems (EM) showing the explored regions during: (A) Titration with ultra-pure water; (B) Titration with copaiba oil-resin (CO); (C) Titration with Soluplus (SOL); (D) Final diagram with all formulations. Figure S2. Titration with ultra-purified water. Diagram showing the representation of the sequence of additions and examples in images of some additions made. The number representing the addition performed is displayed at the bottom of each image. Figure S3. Titration with copaiba oil-resin (CO). Diagram showing the representation of the sequence of additions and examples in images of some additions made. The number representing the addition performed is displayed at the bottom of each image. Figure S4. Titration with Soluplus (SOL). Diagram showing the representation of the sequence of additions and examples in images of some additions made. The number representing the addition performed is displayed at the bottom of each image. Table S3. Consistency classification of emulsions obtained during the water titration. Table S4. Consistency classification of emulsions obtained during the copaiba oil-resin (CO) titration. Table S5. Consistency classification of emulsions obtained during the Soluplus® titration. Figure S5. Ternary phase diagram of self-emulsifying drug delivery systems (SEDDS) showing the explored regions during: (A) Titration with PEG400; (B) Titration with copaiba oil-resin (CO); (C) Titration with Soluplus (SOL); (D) Final diagram with all formulations. Figure S6. Titration with polyethylene glycol 400 (PEG400) for self-emulsifying drug delivery systems (SEDDS). Diagram showing the representation of the sequence of additions and examples in images of some additions made. The number representing the addition performed is displayed at the bottom of each image. Figure S7. Titration with Soluplus (SOL) for self-emulsifying drug delivery systems (SEDDS). Diagram showing the representation of the sequence of additions and examples in images of some additions made. The number representing the addition performed is displayed at the bottom of each image. Figure S8. Titration with copaiba oil-resin (CO) for self-emulsifying drug delivery systems (SEDDS). Diagram showing the representation of the sequence of additions and examples in images of some additions made. The number representing the addition performed is displayed at the bottom of each image. Table S6. Consistency classification of self-emulsifying drug delivery systems obtained during the polyethylene glycol 400 (PEG400) titration. Table S7. Consistency classification of self-emulsifying drug delivery systems obtained during the Soluplus (SOL) titration. Table S8. Consistency classification of self-emulsifying drug delivery systems obtained during the copaiba oil-resin (CO) titration. Figure S9. Location in the diagrams of the formulations selected for the centrifuging tests: (A) Emulsion systems (EM); (B) Self-emulsifying systems (SEDDS). Table S9. Composition of emulsion (EM) and self-emulsifying drug delivery systems (SEDDS) containing copaiba oil-resin (CO), Soluplus (SOL), polyethylene glycol 400 (PEG 400) submitted to centrifuging test. Figure S10. Frequency of distribution of the diameter of the droplets found in emulsifying systems E1 and F14, diluted in ultrapure water, at a temperature of 25 and 37 °C. Result expressed in number of distribution of the mean of three analyzes with coefficient of variation less than 10%.
