# Supplementary material for: Development of Environmentally Responsive Self-Emulsifying System Containing Copaiba Oil-Resin for Leishmaniasis Oral Treatment
Source: Pharmaceutics. 2023 Aug 12;15(8):2127. doi: 10.3390/pharmaceutics15082127 (PMC10459651; doi:10.3390/pharmaceutics15082127)
Supplement: Supplementary file 1 [file pharmaceutics-15-02127-s001.zip › pharmaceutics-2531233-supplementary.pdf]

pharmaceutics

Supplementary Materials: Development of Environmentally Responsive Self-Emulsifying System Containing Copaiba Oil-Resin for Leishmaniasis Oral Treatment

Mariana Carla de Oliveira, Rodolfo Bento Balbinot, Mônica Villa Nova, Renato Sonchini Gonçalves, Danielle Lazarin Bidóia, Wilker Caetano, Celso Vataru Nakamura and Marcos Luciano Bruschi

1. Discussion About the EM Ternary Phase Diagrams

During the titration, it was possible to observe that the aqueous titration (Figure S2 and Table S3) was the one that most caused phase separations of EM. This can be explained by the fact that most of the formulations explored in this diagram region have had an insufficient amount of water for EM formation. The same was observed in relation to CO titration (Figure S3 and Table S4), where the diagram regions that have shown phase separation because of the insufficient amount of water. During the SOL titration (Figure S4 and Table S5), the diagram regions that did not show emulsion corresponded to those with low amounts of surfactant and water.

2. Discussion About the SEDDS Ternary Phase Diagrams

According to the Figure S5, the titration with PEG 400 (Figure S6 and Table S6) has shown more phase separation, especially when there was not sufficient proportion of PEG 400 to disperse the SOL; however, the titration with SOL (Figure S7 and Table S7) displayed less phase separation. During the CO titration (Figure S8 and Table S8), there was phase separation in last titrations (7O, 8O and 9O). Again, this can be explained by the proportion of PEG 400 to disperse the surfactant. Therefore, one of the main factors for the success in the SEDDS development was the SOL and PEG 400 proportions.

Table S1. Scheme of titrations performed to obtain the ternary phase diagram of self-emulsifying drug delivery systems (SEDDS) composed of copaiba oil-resin (CO), Soluplus (SOL) and polyethylene glycol 400 (PEG 400).

**PEG400 TITRATION**		
**Titration**	**Ratio of SOL:CO**	**PEG400 addition**
**1P**	**1:9**	**Additions of PEG 400 up to 33% (*w/w*) of PEG 400**
**2P**	**2:8**	Additions of PEG 400 up to 33% (*w/w*) of PEG 400
**3P**	**3:7**	Additions of PEG 400 up to 33% (*w/w*) of PEG 400
**4P**	**4:6**	Additions of PEG 400 up to 33% (*w/w*) of PEG 400
**5P**	**5:5**	Additions of PEG 400 up to 33% (*w/w*) of PEG 400
**6P**	**6:4**	Additions of PEG 400 up to 33% (*w/w*) of PEG 400
**7P**	**7:3**	Additions of PEG 400 up to 33% (*w/w*) of PEG 400
**8P**	**8:2**	Additions of PEG 400 up to 33% (*w/w*) of PEG 400
**9P**	**9:1**	Additions of PEG 400 up to 33% (*w/w*) of PEG 400
**CO TITRATION**		
**Titration**	**Ratio of SOL:PEG 400**	**CO addition**
**1O**	**1:9**	**Additions of CO up to 33% (*w/w*) of CO**
**2O**	**2:8**	Additions of CO up to 33% (*w/w*) of CO
**3O**	**3:7**	Additions of CO up to 33% (*w/w*) of CO
**4O**	**4:6**	Additions of CO up to 33% (*w/w*) of CO
**5O**	**5:5**	Additions of CO up to 33% (*w/w*) of CO
**6O**	**6:4**	Additions of CO up to 33% (*w/w*) of CO
**7O**	**7:3**	Additions of CO up to 33% (*w/w*) of CO
**8O**	**8:2**	Additions of CO up to 33% (*w/w*) of CO
**9O**	**9:1**	Additions of CO up to 33% (*w/w*) of CO
SOL TITRATION		
**Titration**	**Ratio of CO:PEG 400**	**SOL addition**
**1S**	**1:9**	**Additions of SOL up to 33% (*w/w*) of SOL**
**2S**	**2:8**	Additions of SOL up to 33% (*w/w*) of SOL
**3S**	**3:7**	Additions of SOL up to 33% (*w/w*) of SOL
**4S**	**4:6**	Additions of SOL up to 33% (*w/w*) of SOL
**5S**	**5:5**	Additions of SOL up to 33% (*w/w*) of SOL
**6S**	**6:4**	Additions of SOL up to 33% (*w/w*) of SOL
**7S**	**7:3**	Additions of SOL up to 33% (*w/w*) of SOL
**8S**	**8:2**	Additions of SOL up to 33% (*w/w*) of SOL
**9S**	**9:1**	Additions of SOL up to 33% (*w/w*) of SOL

Table S2. Scheme of titrations performed to obtain the ternary phase diagram of emulsion systems (EM) composed of copaiba oil-resin (CO), Soluplus (SOL) and ultra-pure water.

WATER TITRATION		
**Titration**	**Ratio of SOL:CO**	**Water addition**
**1A**	**1:9**	**Additions of water up to 33% (*w/w*) of water**
**2A**	**2:8**	Additions of water up to 33% (*w/w*) of water
**3A**	**3:7**	Additions of water up to 33% (*w/w*) of water
**4A**	**4:6**	Additions of water up to 33% (*w/w*) of water
**5A**	**5:5**	Additions of water up to 33% (*w/w*) of water
**6A**	**6:4**	Additions of water up to 33% (*w/w*) of water
**7A**	**7:3**	Additions of water up to 33% (*w/w*) of water
**8A**	**8:2**	Additions of water up to 33% (*w/w*) of water
**9A**	**9:1**	Additions of water up to 33% (*w/w*) of water
**CO TITRATION**		
**Titration**	**Ratio of SOL:WATER**	**CO addition**
**1O**	**1:9**	**Additions of CO up to 33% (*w/w*) of CO**
**2O**	**2:8**	Additions of CO up to 33% (*w/w*) of CO
**3O**	**3:7**	Additions of CO up to 33% (*w/w*) of CO
**4O**	**4:6**	Additions of CO up to 33% (*w/w*) of CO
**5O**	**5:5**	Additions of CO up to 33% (*w/w*) of CO
**6O**	**6:4**	Additions of CO up to 33% (*w/w*) of CO
**7O**	**7:3**	Additions of CO up to 33% (*w/w*) of CO
**8O**	**8:2**	Additions of CO up to 33% (*w/w*) of CO
**9O**	**9:1**	Additions of CO up to 33% (*w/w*) of CO
SOL TITRATION		
**Titration**	**Ratio of CO:WATER**	**Soluplus® addition**
**1T**	**1:9**	**Additions of SOL up to 33% (*w/w*) of SOL**
**2T**	**2:8**	Additions of SOL up to 33% (*w/w*) of SOL
**3T**	**3:7**	Additions of SOL up to 33% (*w/w*) of SOL
**4T**	**4:6**	Additions of SOL up to 33% (*w/w*) of SOL
**5T**	**5:5**	Additions of SOL up to 33% (*w/w*) of SOL
**6T**	**6:4**	Additions of SOL up to 33% (*w/w*) of SOL
**7T**	**7:3**	Additions of SOL up to 33% (*w/w*) of SOL
**8T**	**8:2**	Additions of SOL up to 33% (*w/w*) of SOL
**9T**	**9:1**	Additions of SOL up to 33% (*w/w*) of SOL

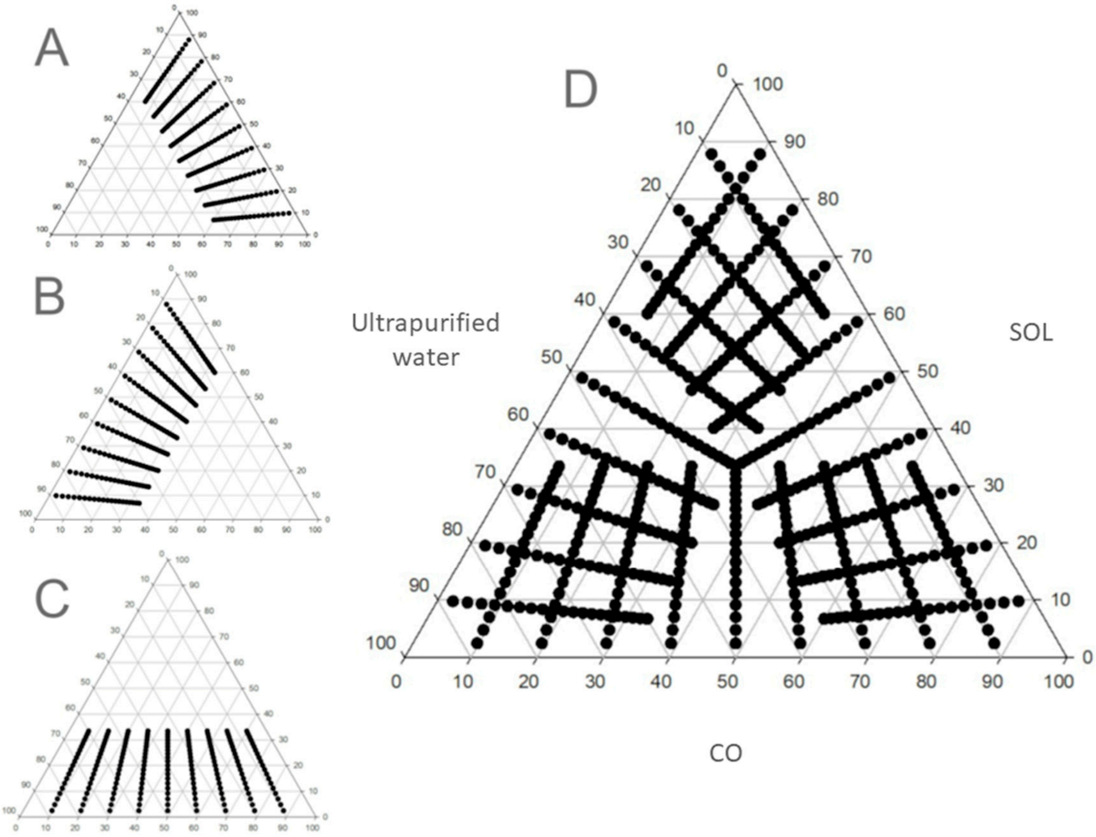

Figure S1. Ternary phase diagrams of emulsion systems (EM) showing the explored regions during: (**A**) Titration with ultra-pure water; (**B**) Titration with copaiba oil-resin (CO); (**C**) Titration with Soluplus (SOL); (**D**) Final diagram with all formulations.

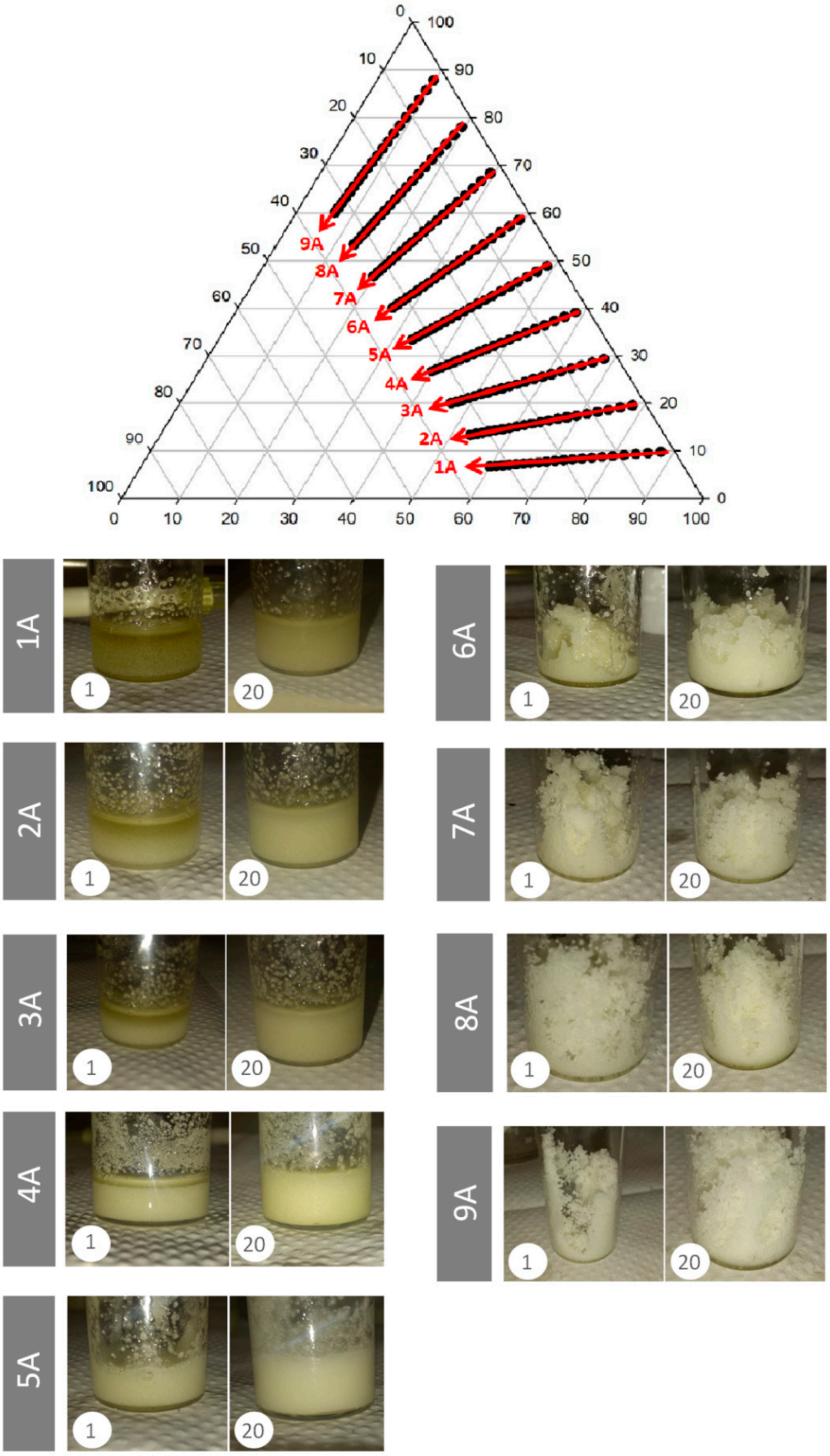

Figure S2. Titration with ultra-purified water. Diagram showing the representation of the sequence of additions and examples in images of some additions made. The number representing the addition performed is displayed at the bottom of each image.

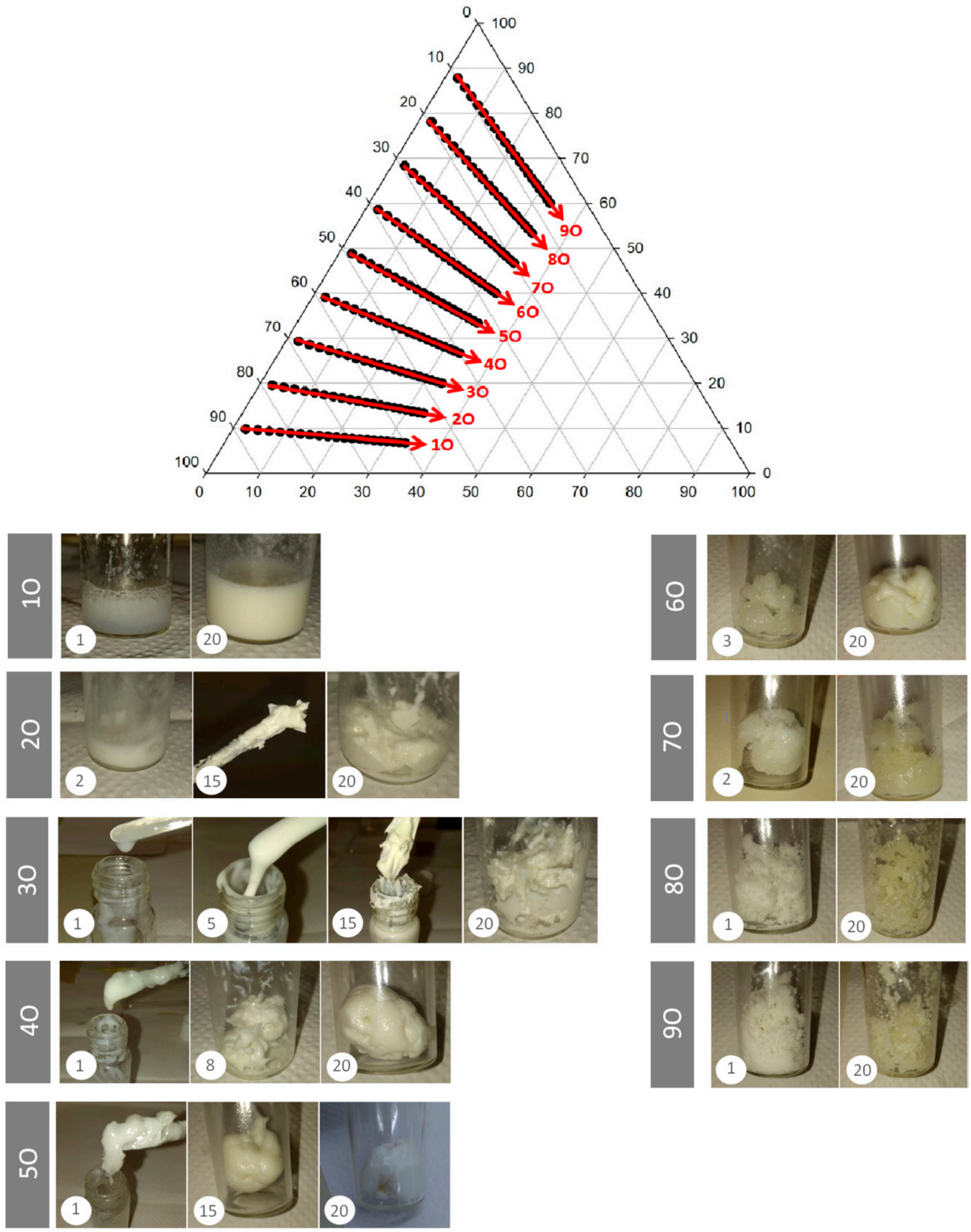

Figure S3. Titration with copaiba oil-resin (CO). Diagram showing the representation of the sequence of additions and examples in images of some additions made. The number representing the addition performed is displayed at the bottom of each image.

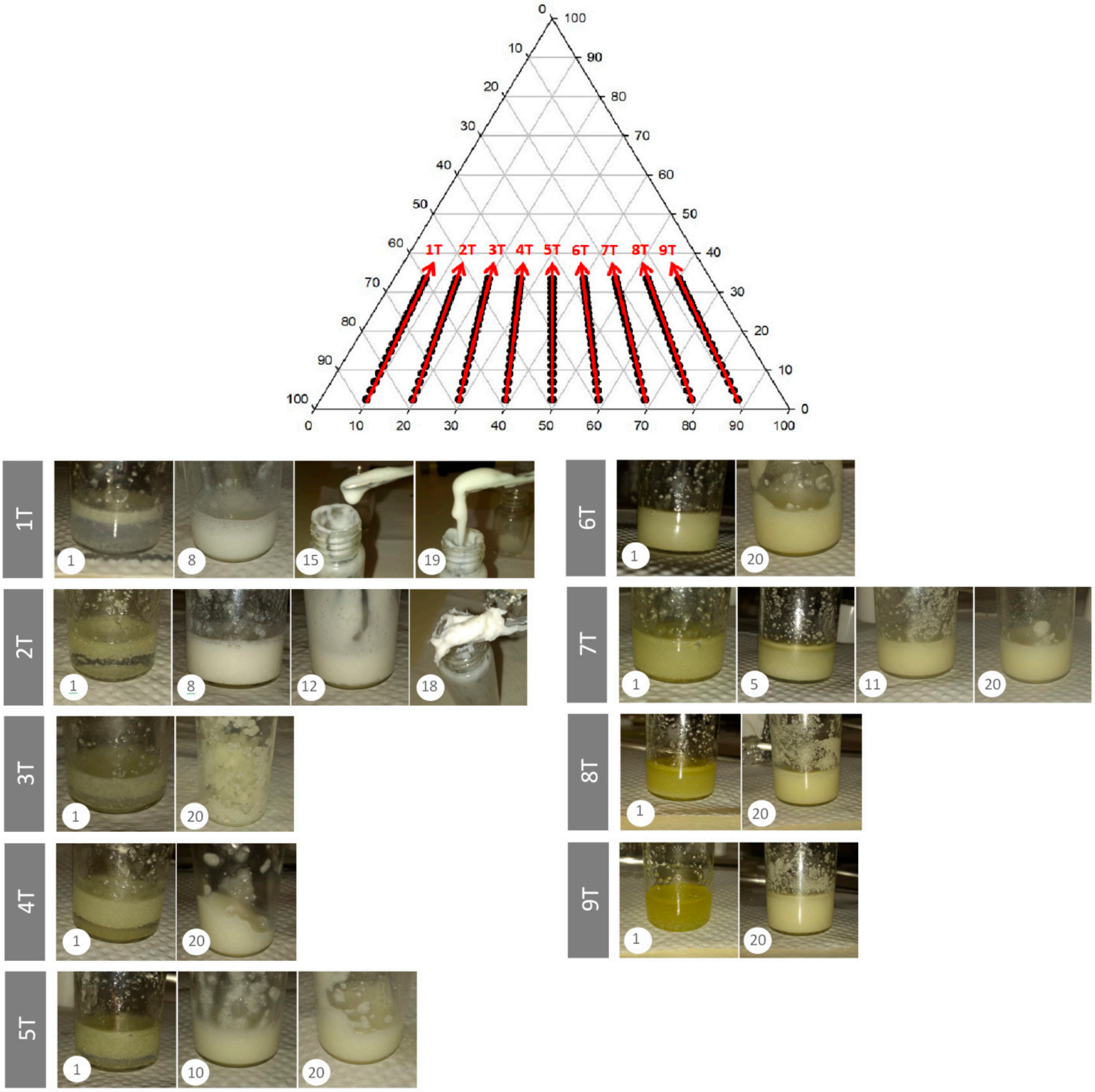

Figure S4. Titration with Soluplus (SOL). Diagram showing the representation of the sequence of additions and examples in images of some additions made. The number representing the addition performed is displayed at the bottom of each image.

Table S3. Consistency classification of emulsions obtained during the water titration.

Titration	SOL and CO		Ultra-purified water (WATER ADDITION)																			
	Ratio of SOL:OC	Weight (g)	1	2	3	4	5	6	7	8	9	10	11	12	13	14	15	16	17	18	19	20
			50 µg	100 µg	150 µg	200 µg	250 µg	300 µg	350 µg	400 µg	450 µg	500 µg	550 µg	600 µg	650 µg	700 µg	750 µg	800 µg	850 µg	900 µg	950 µg	1000 µg
1A	1:9	SOL: 0.2 CO: 1.8	PS	PS	PS	PS	PS	PS	PS	PS	PS	PS	PS	PS	PS	PS	PS	PS	PS	PS	PS	PS
2A	2:8	SOL: 0.4 CO: 1.6	PS	PS	PS	PS	PS	PS	PS	PS	PS	PS	PS	PS	PS	PS	PS	PS	PS	PS	PS	LE
3A	3:7	SOL: 0.6 CO: 1.4	PS	PS	PS	PS	PS	PS	PS	PS	PS	PS	PS	PS	PS	PS	PS	PS	PS	LE	LE	LE
4A	4:6	SOL: 0.8 CO: 1.2	PS	PS	PS	PS	PS	PS	PS	PS	PS	PS	PS	PS	PS	PS	LE	LE	LE	LE	LE	LE
5A	5:5	SOL: 1.0 CO: 1.0	PS	PS	PS	PS	PS	PS	PS	PS	PS	LE	LE	LE	LE	LE	LE	LE	LE	LE	LE	LE
6A	6:4	SOL: 1.2 CO: 0.8	PS	PS	PS	PS	PS	PS	PS	PS	PS	PS	VE +++	VE +++	VE +++	VE +++	VE +++	VE +++	VE +++	VE +++	VE +++	VE +++
7A	7:3	SOL: 1.4 CO: 0.6	PS	PS	PS	PS	PS	PS	PS	PS	PS	PS	VE +++	VE +++	VE +++	VE +++	VE +++	VE +++	VE +++	VE +++	VE +++	VE +++
8A	8:2	SOL: 1.6 CO: 0.4	PS	PS	PS	PS	PS	PS	PS	PS	PS	PS	PS	PS	VE +++	VE +++	VE +++	VE +++	VE +++	VE +++	VE +++	VE +++
9A	9:1	SOL: 1.8 CO: 0.2	PS	PS	PS	PS	PS	PS	PS	PS	PS	PS	PS	PS	PS	VE +++	VE +++	VE +++	VE +++	VE +++	VE +++	VE +++

PS = Phase separation; LE = Liquid emulsion; VE+ = Viscous emulsion +; VE++ = Viscous emulsion ++; VE+++ = Viscous emulsion +++.

Table S4. Consistency classification of emulsions obtained during the copaiba oil-resin (CO) titration**.**

CO ADDITION																						
Titration	SOL and WATER		CO																			
	Ratio of SOL:WATER	Weight (g)	1	2	3	4	5	6	7	8	9	10	11	12	13	14	15	16	17	18	19	20
			50 µg	100 µg	150 µg	200 µg	250 µg	300 µg	350 µg	400 µg	450 µg	500 µg	550 µg	600 µg	650 µg	700 µg	750 µg	800 µg	850 µg	900 µg	950 µg	1000 µg
1O	1:9	SOL: 0.2 WATER: 1.8	PS	PS	PS	LE	LE	LE	LE	LE	LE	LE	LE	LE	LE	LE	LE	LE	LE	LE	LE	LE
2O	2:8	SOL: 0.4 WATER: 1.6	LE	LE	LE	LE	LE	LE	LE	VE +	VE +	VE +	VE +	VE +	VE +	VE +	VE +	VE +	VE ++	VE ++	VE ++	VE ++
3O	3:7	SOL: 0.6 WATER: 1.4	LE	LE	LE	LE	LE	LE	LE	LE	LE	LE	LE	LE	VE +	VE +	VE +	VE +	VE +	VE +	VE +	VE+
4O	4:6	SOL: 0.8 WATER: 1.2	VE +	VE +	VE ++	VE ++	VE ++	VE ++	VE ++	VE ++	VE ++	VE ++	VE +++	VE +++	VE +++	VE +++	VE +++	VE +++	VE +++	VE +++	VE +++	VE +++
5O	5:5	SOL: 1.0 WATER : 1.0	VE ++	VE ++	VE ++	VE ++	VE ++	VE +++	VE +++	VE +++	VE +++	VE +++	VE +++	VE +++	VE +++	VE +++	VE +++	VE +++	VE +++	VE +++	EL	EL
6O	6:4	SOL: 1.2 WATER : 0.8	VE ++	VE ++	VE ++	VE ++	VE +++	VE +++	VE +++	VE +++	VE +++	VE +++	VE +++	VE +++	VE +++	VE +++	VE +++	VE +++	VE +++	VE +++	VE +++	VE +++
7O	7:3	SOL: 1.4 WATER : 0.6	VE +++	VE +++	VE +++	VE +++	VE +++	VE +++	VE +++	VE +++	VE +++	VE +++	VE +++	VE +++	VE +++	VE +++	VE +++	VE +++	VE +++	VE +++	VE +++	VE +++
8O	8:2	SOL: 1.6 WATER : 0.4	PS	PS	PS	PS	PS	PS	PS	PS	PS	PS	PS	PS	PS	PS	PS	PS	PS	PS	PS	PS
9O	9:1	SOL: 1.8 WATER : 0.2	PS	PS	PS	PS	PS	PS	PS	PS	PS	PS	PS	PS	PS	PS	PS	PS	PS	PS	PS	PS

PS = Phase separation; LE = Liquid emulsion; VE+ = Viscous emulsion +; VE++ = Viscous emulsion ++; VE+++ = Viscous emulsion +++.

Table S5. Consistency classification of emulsions obtained during the Soluplus® titration.

	SOL ADDITION																				
Titration	CO and WATER		SOL																		
	Ratio of CO:WATER	Weight (g)	1	2	3	4	5	6	7	8	9	10	11	12	13	14	15	16	17	18	19	20
			50 µg	100 µg	150 µg	200 µg	250 µg	300 µg	350 µg	400 µg	450 µg	500 µg	550 µg	600 µg	650 µg	700 µg	750 µg	800 µg	850 µg	900 µg	950 µg	1000 µg
1T	1:9	CO: 0.2 WATER: 1.8	PS	PS	PS	LE	LE	LE	LE	LE	LE	LE	VE +	VE +	VE +	VE +	VE +	VE +	VE +	VE +	VE +	VE +
2T	2:8	CO : 0.4 WATER: 1.6	PS	PS	LE	LE	LE	LE	LE	VE +	VE +	VE +	VE +	VE +	VE +	VE +	VE ++	VE ++	VE ++	VE ++	VE ++	VE ++
3T	3:7	CO : 0.6 WATER: 1.4	PS	PS	LE	LE	LE	LE	VE +	VE +	VE +	VE +	VE +	VE +	VE ++	VE ++	VE ++	VE ++	VE ++	VE ++	VE +++	VE +++
4T	4:6	CO : 0.8 WATER: 1.2	PS	PS	LE	LE	LE	VE ++	VE ++	VE ++	VE ++	VE ++	VE ++	VE ++	VE ++	VE ++	VE ++	VE +++	VE +++	VE +++	VE +++	VE +++
5T	5:5	CO : 1.0 WATER : 1.0	LE	LE	LE	LE	LE	LE	LE	LE	LE	LE	LE	LE	LE	LE	LE	LE	LE	LE	LE	LE
6T	6:4	CO : 1.2 WATER : 0.8	LE	LE	LE	LE	LE	LE	LE	LE	LE	LE	LE	LE	LE	LE	LE	LE	LE	LE	LE	LE
7T	7:3	CO : 1.4 WATER : 0.6	PS	PS	PS	PS	PS	PS	PS	PS	PS	PS	PS	PS	PS	PS	PS	PS	PS	PS	PS	PS
8T	8:2	CO : 1.6 WATER : 0.4	PS	PS	PS	PS	PS	PS	PS	PS	PS	PS	PS	PS	PS	PS	PS	PS	PS	PS	PS	PS
9T	9:1	CO : 1.8 WATER : 0.2	PS	PS	PS	PS	PS	PS	PS	PS	PS	PS	PS	PS	PS	PS	PS	PS	PS	PS	PS	PS

PS = Phase separation; LE = Liquid emulsion; VE+ = Viscous emulsion +; VE++ = Viscous emulsion ++; VE+++ = Viscous emulsion +++.

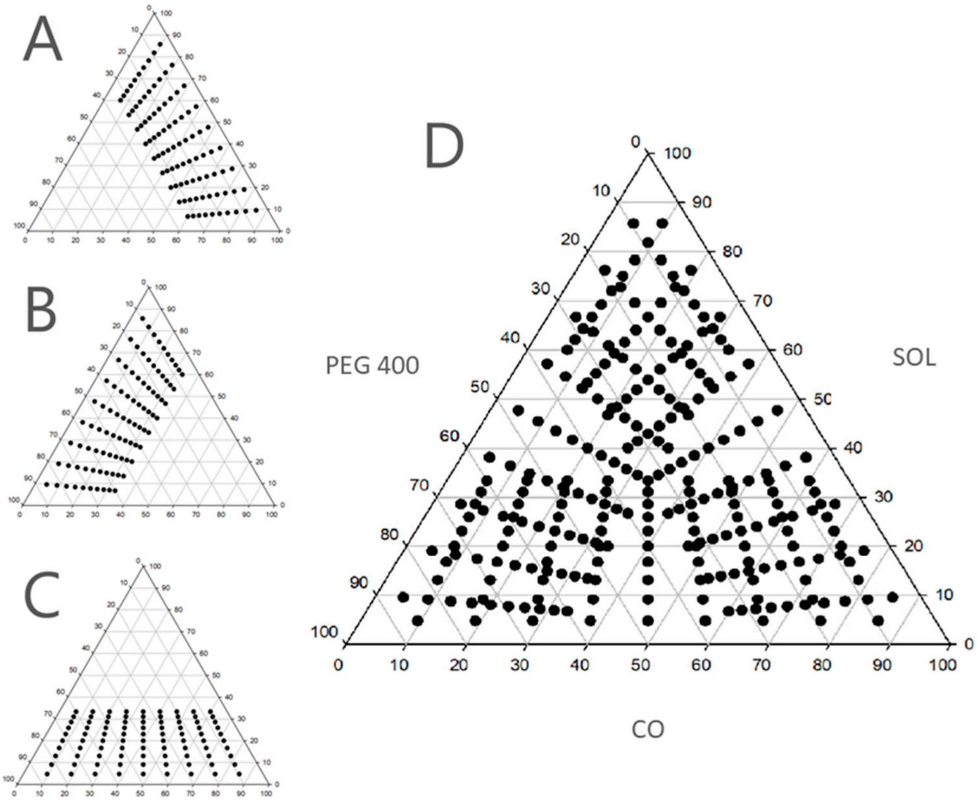

Figure S5. Ternary phase diagram of self-emulsifying drug delivery systems (SEDDS) showing the explored regions during: (**A**) Titration with PEG400; (**B**) Titration with copaiba oil-resin (CO); (**C**) Titration with Soluplus (SOL); (**D**) Final diagram with all formulations.

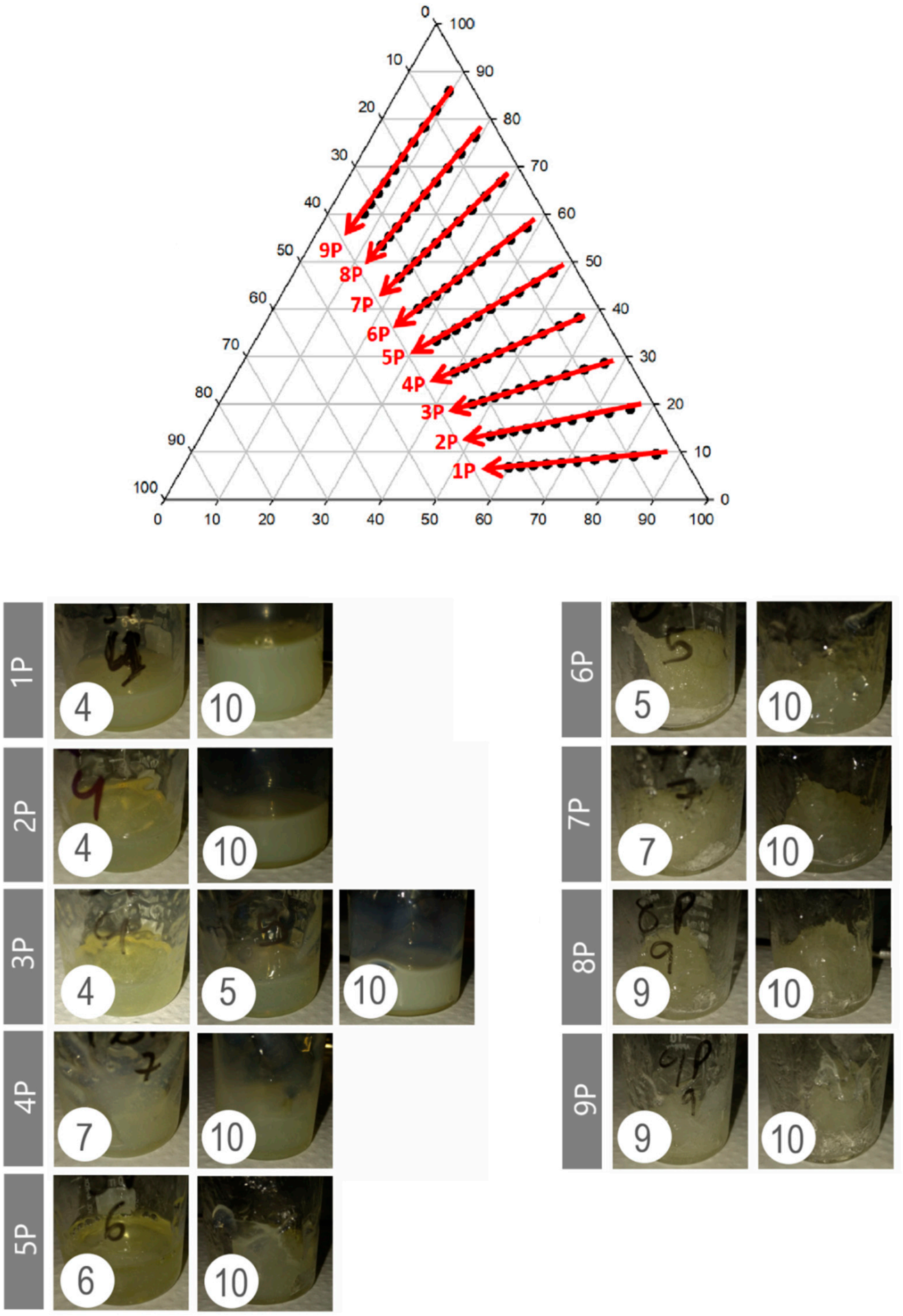

Figure S6. Titration with polyethylene glycol 400 (PEG400) for self-emulsifying drug delivery systems (SEDDS). Diagram showing the representation of the sequence of additions and examples in images of some additions made. The number representing the addition performed is displayed at the bottom of each image.

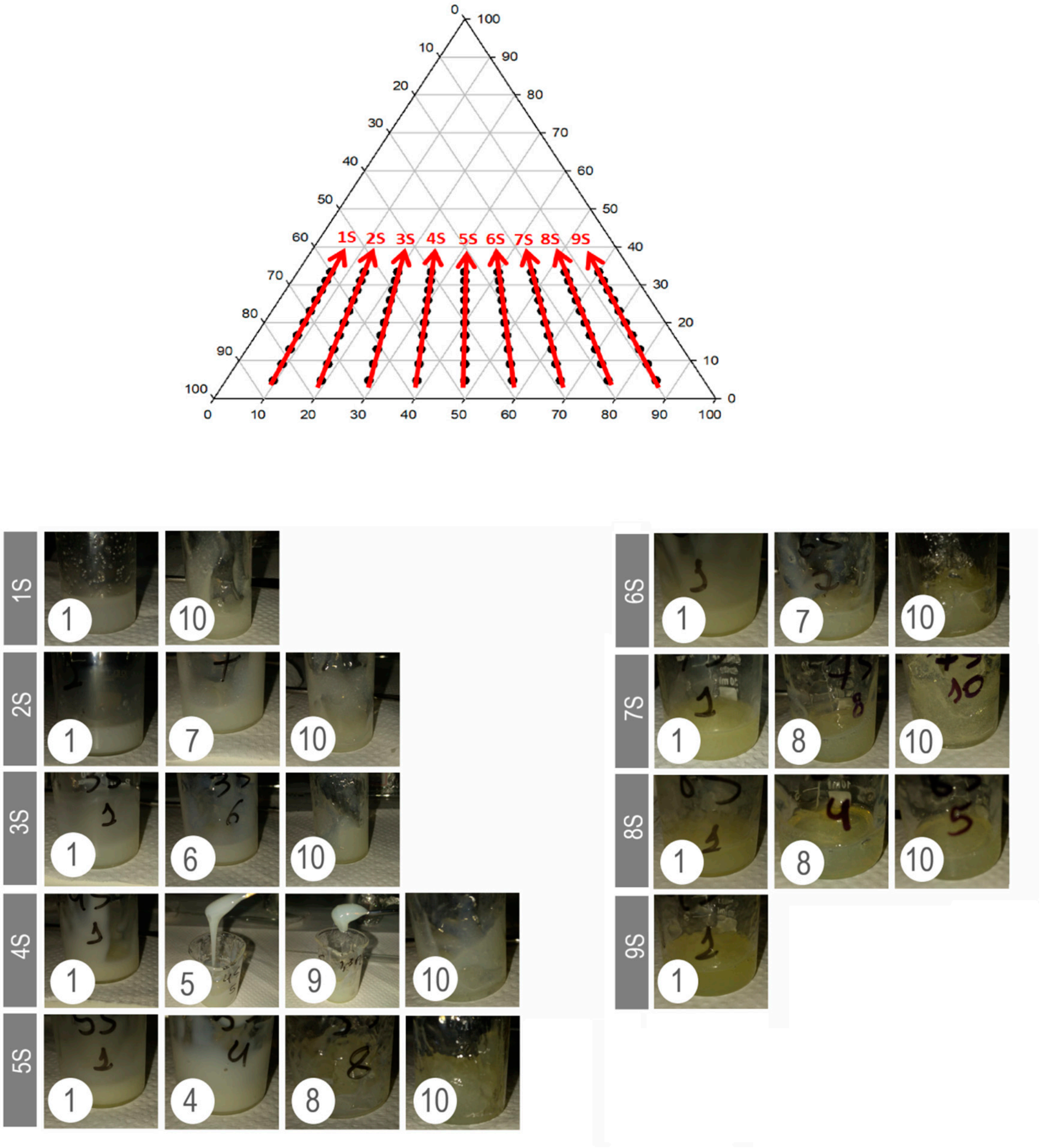

Figure S7. Titration with Soluplus (SOL) for self-emulsifying drug delivery systems (SEDDS). Diagram showing the representation of the sequence of additions and examples in images of some additions made. The number representing the addition performed is displayed at the bottom of each image.

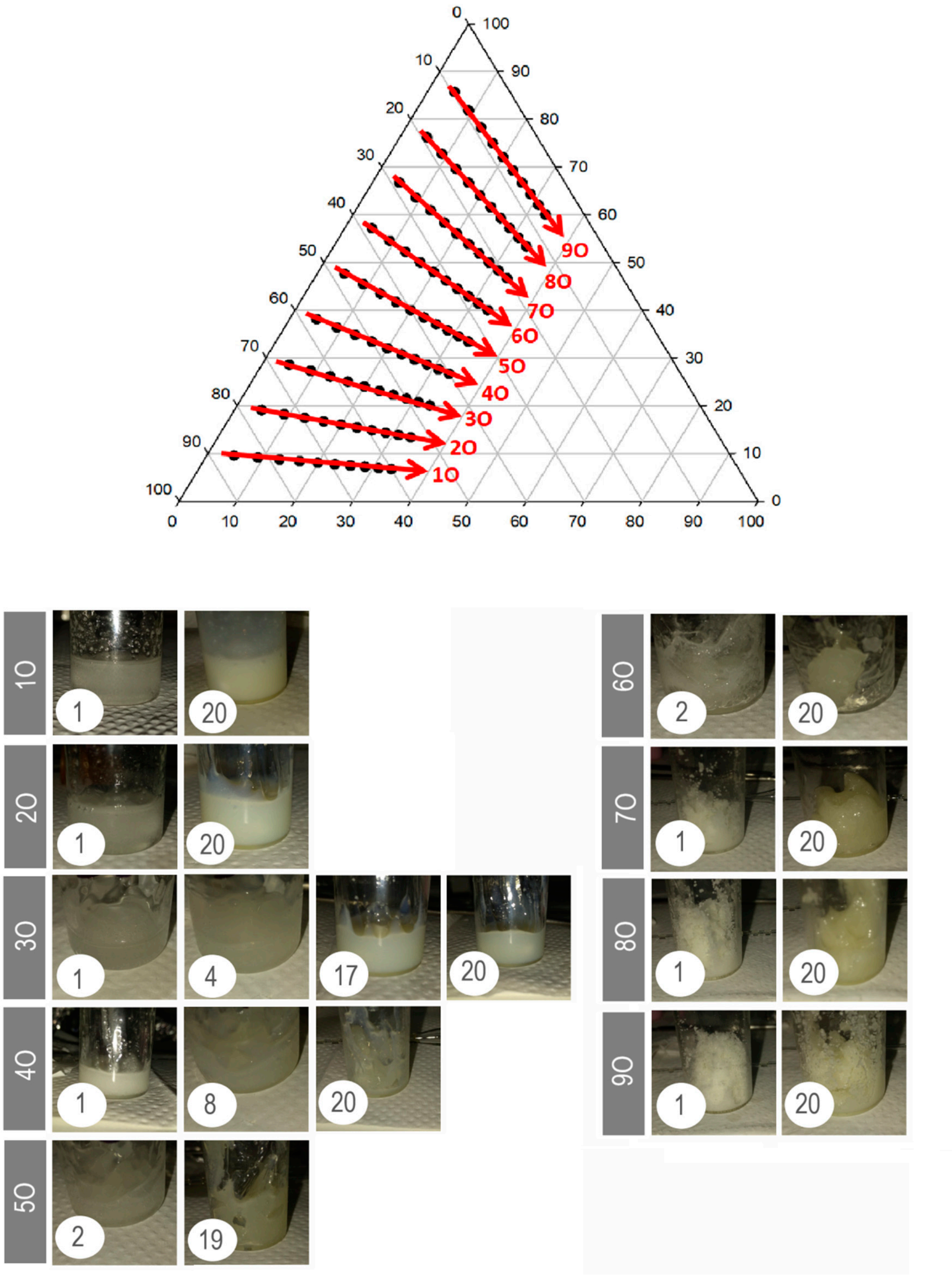

Figure S8. Titration with copaiba oil-resin (CO) for self-emulsifying drug delivery systems (SEDDS). Diagram showing the representation of the sequence of additions and examples in images of some additions made. The number representing the addition performed is displayed at the bottom of each image.

Table S6. Consistency classification of self-emulsifying drug delivery systems obtained during the polyethylene glycol 400 (PEG400) titration.

PEG 400 ADDITION												
Titration	SOL and CO		PEG 400									
	Ratio of SOL:CO	Weight (g)	1<br>100 µg	2<br>200 µg	3<br>300 µg	4<br>400 µg	5<br>500 µg	6<br>600 µg	7<br>700 µg	8<br>800 µg	9<br>900 µg	10<br>1000 µg
1P	1:9	SOL: 0.2<br>CO: 1.8	PS	PS	L	L	L	L	L	L	L	L
2P	2:8	SOL: 0.4<br>CO: 1.6	PS	PS	PS	L	L	L	L	L	L	L
3P	3:7	SOL: 0.6<br>CO: 1.4	PS	PS	PS	PS	V+	V+	V+	L	L	L
4P	4:6	SOL: 0.8<br>CO: 1.2	PS	PS	PS	PS	PS	V++	V++	V++	V++	V+
5P	5:5	SOL: 1.0<br>CO: 1.0	PS	PS	PS	PS	PS	PS	V+++	V+++	V+++	V+++
6P	6:4	SOL: 1.2<br>CO: 0.8	PS	PS	PS	PS	PS	V +++	V+++	V+++	V+++	V+++
7P	7:3	SOL: 1.4<br>CO: 0.6	PS	PS	PS	PS	PS	PS	PS	V+++	V+++	V+++
8P	8:2	SOL: 1.6<br>CO: 0.4	PS	PS	PS	PS	PS	PS	PS	PS	V+++	V+++
9P	9:1	SOL: 1.8<br>CO: 0.2	PS	PS	PS	PS	PS	PS	PS	PS	PS	PS

PS = Phase separation; L = Liquid; V+ = Viscous +; V++ = Viscous ++; V+++ = Viscous +++.

Table S7. Consistency classification of self-emulsifying drug delivery systems obtained during the Soluplus (SOL) titration.

SOL ADDITION												
Titration	SOL and CO		SOL									
	Ratio of CO:PEG 400	Weight (g)	1<br>100 µg	2<br>200 µg	3<br>300 µg	4<br>400 µg	5<br>500 µg	6<br>600 µg	7<br>700 µg	8<br>800 µg	9<br>900 µg	10<br>1000 µg
1S	1:9	CO: 0.2<br>PEG 400: 1.8	L	L	L	L	L	V+	V+	V+	V+	V+
2S	2:8	CO: 0.4<br>PEG 400: 1.6	L	L	L	L	L	V+	V+	V+	V++	V++
3S	3:7	CO: 0.6<br>PEG 400: 1.4	L	L	L	L	L	V+	V+	V+	V++	V++
4S	4:6	CO: 0.8<br>PEG 400: 1.2	L	L	L	L	V+	V+	V+	V+	V++	V++
5S	5:5	CO: 1.0<br>PEG 400: 1.0	L	L	L	V+	V+	V++	V++	V++	V++	V+++
6S	6:4	CO: 1.2<br>PEG 400: 0.8	L	L	L	L	L	L	V+	V++	V++	PS
7S	7:3	CO: 1.4<br>PEG 400: 0.6	L	L	L	L	L	V+	V+	V+	V++	V++
8S	8:2	CO: 1.6<br>PEG 400: 0.4	L	L	L	L	V+	V+	V+	V+	V++	V++
9S	9:1	CO: 1.8<br>PEG 400: 0.2	PS	PS	PS	PS	PS	PS	PS	PS	PS	PS

PS = Phase separation; L = Liquid; V+ = Viscous +; V++ = Viscous ++; V+++ = Viscous +++.

Table S8. Consistency classification of self-emulsifying drug delivery systems obtained during the copaiba oil-resin (CO) titration.

CO ADDITION												
Titration	SOL and PEG 400		COPAIBA OLEORESIN									
	Ratio of SOL:PEG 400	Weight (g)	1<br>100 µg	2<br>200 µg	3<br>300 µg	4<br>400 µg	5<br>500 µg	6<br>600 µg	7<br>700 µg	8<br>800 µg	9<br>900 µg	10<br>1000 µg
1O	1:9	SOL: 0.2<br>PEG 400: 1.8	L	L	L	L	L	L	L	L	L	PS
2O	2:8	SOL: 0.4<br>PEG 400: 1.6	L	L	L	L	L	L	L	L	L	L
3O	3:7	SOL: 0.6<br>PEG 400: 1.4	L	V+	V+	V+	V+	V+	V+	V+	V++	V++
4O	4:6	SOL: 0.8<br>PEG 400: 1.2	V+	V+	V+	V++	V++	V++	V++	V++	V++	V++
5O	5:5	SOL: 1.0<br>PEG 400: 1.0	V++	V++	V++	V++	V++	V++	V++	V++	V+++	V+++
6O	6:4	SOL: 1.2<br>PEG 400: 0.8	V++	V++	V+++	V+++	V+++	V+++	V+++	V+++	V+++	V+++
7O	7:3	SOL: 1.4<br>PEG 400: 0.6	PS	PS	PS	PS	PS	PS	PS	PS	PS	PS
8O	8:2	SOL: 1.6<br>PEG 400: 0.4	PS	PS	PS	PS	PS	PS	PS	PS	PS	PS
9O	9:1	SOL: 1.8<br>PEG 400: 0.2	PS	PS	PS	PS	PS	PS	PS	PS	PS	PS

PS = Phase separation; L = Liquid; V+ = Viscous +; V++ = Viscous ++; V+++ = Viscous +++.

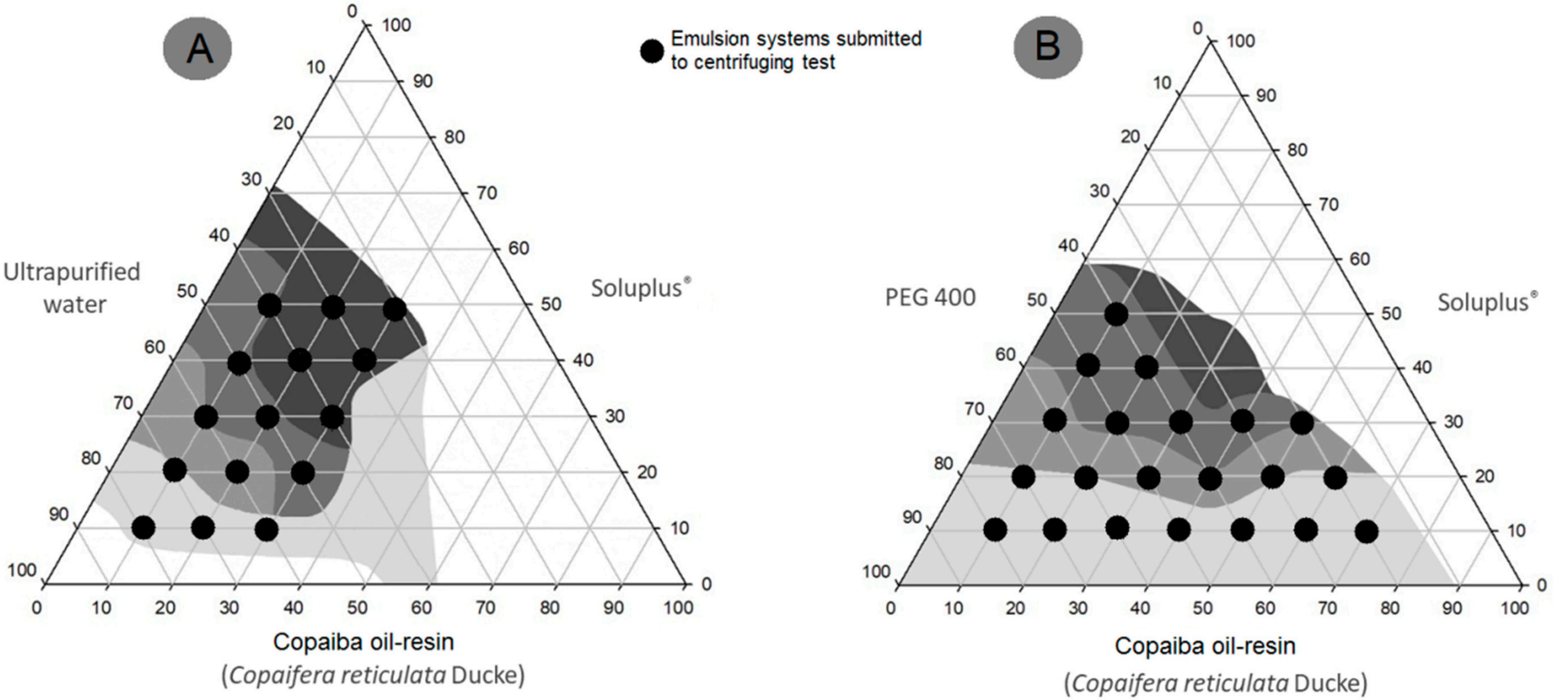

Figure S9. Location in the diagrams of the formulations selected for the centrifuging tests: (**A**) Emulsion systems (EM); (**B**) Self-emulsifying systems (SEDDS).

Table S9. Composition of emulsion (EM) and self-emulsifying drug delivery systems (SEDDS) containing copaiba oil-resin (CO), Soluplus (SOL), polyethylene glycol 400 (PEG 400) submitted to centrifuging test.

Formulation	Type of emulsifying system	Composition (%, *w/w*)			
		Ultra-pure water	CO	SOL	PEG 400
E1	**Emulsion**	60	10	30	-
E2	Emulsion	50	20	30	-
E3	Emulsion	50	10	40	-
E4	Emulsion	80	10	10	-
E5	Emulsion	70	10	20	-
E6	Emulsion	40	10	50	-
E7	Emulsion	70	20	10	-
E8	Emulsion	60	20	20	-
E9	Emulsion	40	20	40	-
E10	Emulsion	30	20	50	-
E11	Emulsion	60	30	10	-
E12	Emulsion	50	30	20	-
E13	Emulsion	40	30	30	-
E14	Emulsion	30	30	40	-
E15	Emulsion	20	30	50	-
F1	**Self-emulsifying**	-	10	10	80
F2	Self-emulsifying	-	20	10	70
F3	Self-emulsifying	-	30	10	60
F4	Self-emulsifying	-	40	10	50
F5	Self-emulsifying	-	50	10	40
F6	Self-emulsifying	-	60	10	30
F7	Self-emulsifying	-	70	10	20
F8	Self-emulsifying	-	10	20	70
F9	Self-emulsifying	-	20	20	60
F10	Self-emulsifying	-	30	20	50
F11	Self-emulsifying	-	40	20	40
F12	Self-emulsifying	-	50	20	30
F13	Self-emulsifying	-	60	20	20
F14	Self-emulsifying	-	10	30	60
F15	Self-emulsifying	-	20	30	50
F16	Self-emulsifying	-	30	30	40
F17	Self-emulsifying	-	40	30	30
F18	Self-emulsifying	-	50	30	20
F19	Self-emulsifying	-	10	40	50
F20	Self-emulsifying	-	20	40	40
F21	Self-emulsifying	-	10	50	40

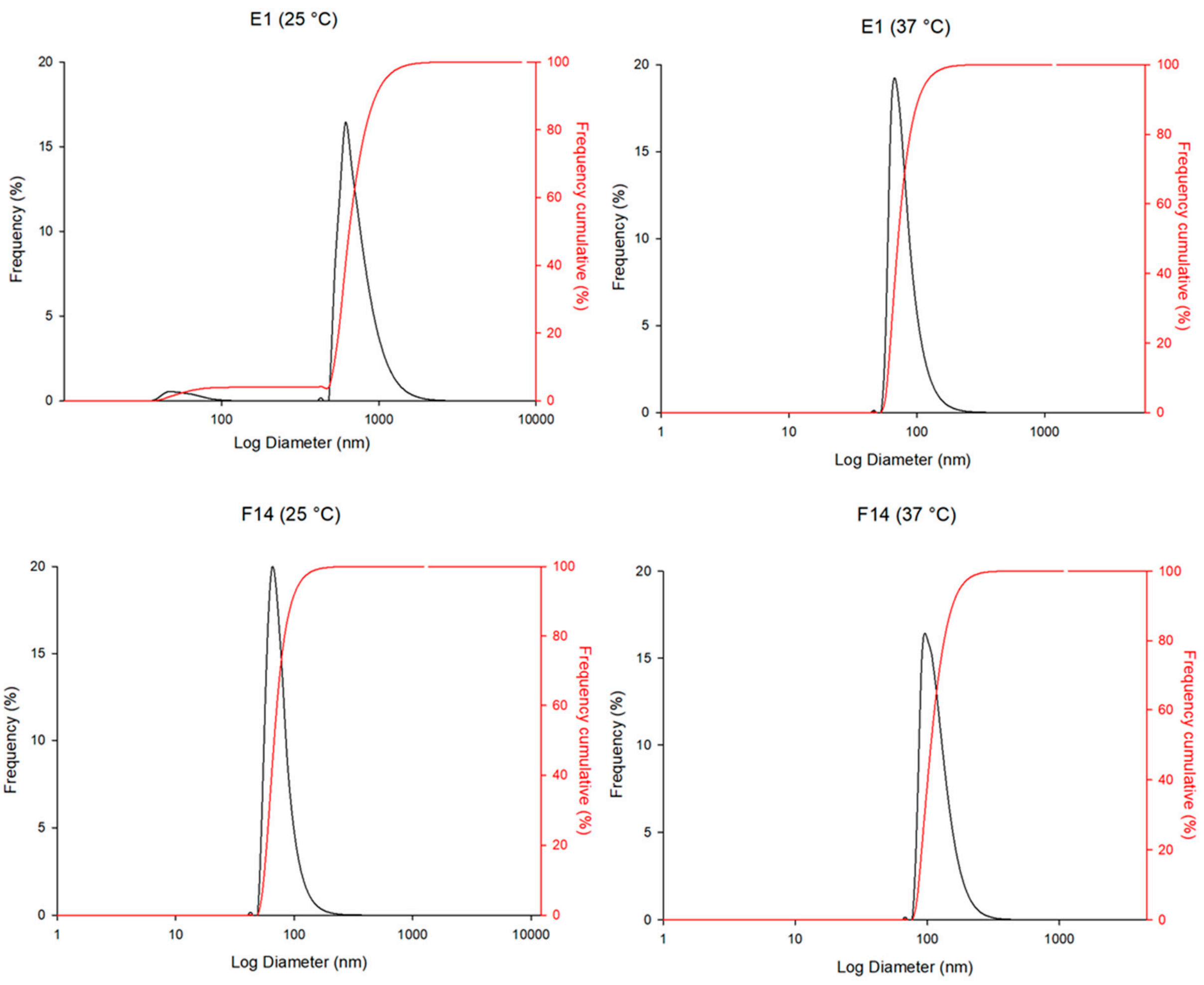

Figure S10. Frequency of distribution of the diameter of the droplets found in emulsifying systems E1 and F14, diluted in ultrapure water, at a temperature of 25 and 37 °C. Result expressed in number of distribution of the mean of three analyzes with coefficient of variation less than 10%.
